# Neurobehavioral Mechanisms of Temporal Processing Deficits in Parkinson's Disease

**DOI:** 10.1371/journal.pone.0017461

**Published:** 2011-02-25

**Authors:** Deborah L. Harrington, Gabriel N. Castillo, Paul A. Greenberg, David D. Song, Stephanie Lessig, Roland R. Lee, Stephen M. Rao

**Affiliations:** 1 Research, Neurology, and Radiology Services, Veterans Affairs San Diego Healthcare System, San Diego, California, United States of America; 2 Department of Radiology, University of California San Diego, San Diego, California, United States of America; 3 Department of Neurosciences, University of California San Diego, San Diego, California, United States of America; 4 Neurological Institute, Cleveland Clinic, Cleveland, Ohio, United States of America; The University of Western Ontario, Canada

## Abstract

**Background:**

Parkinson's disease (PD) disrupts temporal processing, but the neuronal sources of deficits and their response to dopamine (DA) therapy are not understood. Though the striatum and DA transmission are thought to be essential for timekeeping, potential working memory (WM) and executive problems could also disrupt timing.

**Methodology/Findings:**

The present study addressed these issues by testing controls and PD volunteers ‘on’ and ‘off’ DA therapy as they underwent fMRI while performing a time-perception task. To distinguish systems associated with abnormalities in temporal and non-temporal processes, we separated brain activity during encoding and decision-making phases of a trial. Whereas both phases involved timekeeping, the encoding and decision phases emphasized WM and executive processes, respectively. The methods enabled exploration of both the amplitude and temporal dynamics of neural activity. First, we found that time-perception deficits were associated with striatal, cortical, and cerebellar dysfunction. Unlike studies of timed movement, our results could not be attributed to traditional roles of the striatum and cerebellum in movement. Second, for the first time we identified temporal and non-temporal sources of impaired time perception. Striatal dysfunction was found during both phases consistent with its role in timekeeping. Activation was also abnormal in a WM network (middle-frontal and parietal cortex, lateral cerebellum) during encoding and a network that modulates executive and memory functions (parahippocampus, posterior cingulate) during decision making. Third, hypoactivation typified neuronal dysfunction in PD, but was sometimes characterized by abnormal temporal dynamics (e.g., lagged, prolonged) that were not due to longer response times. Finally, DA therapy did not alleviate timing deficits.

**Conclusions/Significance:**

Our findings indicate that impaired timing in PD arises from nigrostriatal and mesocortical dysfunction in systems that mediate temporal and non-temporal control-processes. However, time perception impairments were not improved by DA treatment, likely due to inadequate restoration of neuronal activity and perhaps corticostriatal effective-connectivity.

## Introduction

Timing is a process that helps structure perception, cognition and movement. Prevailing models emphasize the role of the striatum and dopamine (DA) neurotransmission [1], [2] in regulating an internal clock that generates pulses and an accumulator that counts pulses, thereby representing perceived duration. The experience of time, however, can be dilated or compressed by working memory (WM), attention, and decisional processes [3], which are cortically driven. Thus, timing emerges from interactions among multiple processes that are intertwined. When timing is disentangled from other processes, the striatum is closely linked to timing, whereas the supplementary motor area (SMA) and the middle-frontal and inferior parietal cortices are more associated with WM and executive processes, respectively [4].

The basal ganglia's role in timing is particularly relevant to individuals with Parkinson's disease (PD), who exhibit temporal processing deficits [5]–[12]. Timing deficits may contribute to the breakdown in the spatiotemporal patterning of movements in PD, which benefit from external rhythmic sensory-cueing [13]. The neuronal sources of timing impairments in PD and their response to DA therapy are not well understood. Whether DA therapy improves timing deficits is controversial [9]–[12], [14]–[16]. To date, three fMRI and one PET study of timing have been conducted in PD [16]–[19]. Only two of these studies examined the effect of DA treatment [16], [19], and all studied timed movements, so that it was not possible to distinguish abnormal activation in systems classically associated with motor-control (i.e., basal ganglia, cerebellum) from activity related to temporal processing.

The present study addressed these issues by testing PD participants ‘on’ and ‘off’ their DA therapy as they underwent fMRI while performing a time-perception task. In this task, a standard interval (SI) and a comparison interval (CI) were successively encoded, followed by a decision about their relative duration. To identify neural systems related to different components of temporal processing, we separated brain activation associated with encoding the SI and holding it in WM from activation associated with encoding the CI and making a decision. We reasoned that the encoding phase would emphasize timekeeping, but also WM maintenance. Whereas the decision phase engages timekeeping as well, executive processes involved in updating WM and comparing information is also emphasized during this period [4], [20], [21]. We predicted that abnormal basal ganglia activation in PD would be seen during both phases if the striatum is critical for timekeeping. As SMA dysfunction is common in PD, we also expected abnormal activation during both phases if the SMA plays a key role in timekeeping [22]. Finally, we predicted abnormal middle-frontal cortex activation during the decision, but not the encoding phase if executive difficulties [23], [24] contribute to timing deficits in PD. To determine if the cognitive-control systems emphasized by the two phases respond differently to DA therapy [25], we studied the effects of medication on brain activation and on striatal interactions with the cortex and cerebellum (i.e., effective connectivity). Though DA therapy was expected to improve striatal function, its effects on key cortical regions that support time perception (e.g., SMA, middle-frontal and inferior-parietal cortex) are unclear as this has not been previously studied.

## Methods

### Participants and Procedures

Participants included 21 volunteers with idiopathic PD (14 males, 7 females) and 19 healthy adults (12 males, 7 females). Age and education were balanced between the groups (Table 1). Subjects were excluded if they had metal in their body, exhibited signs of dementia on a global dementia screening battery (Mini-Mental Status Exam score <25) and on neuropsychological tests of cognitive speed/flexibility, working memory span, and sustained attention (i.e., ≥1.5 standard deviations (SD) below the control group; Table 1), exhibited signs of depression (Geriatric Depression Score ≥10), or had a medical history of neurological diagnoses other than PD, severe psychiatric disorders (DSM-IV), diabetes, and alcohol or substance abuse. PD volunteers were excluded if they had axial tremors or dyskinesias that could cause head movement during scanning, or if they were taking cholinesterase inhibitors or neuroleptic medications. ANOVAs showed no group differences on neuropsychological tests of global dementia, cognitive speed/flexibility, verbal and spatial working memory, sustained attention and maximum tapping speed (Table 1).

**Table 1:** Demographics, neuropsychological test performance 1, and structural MRI volumes for the Control and Parkinson's groups.

Variables	Control (n = 19)		Parkinson's (n = 21)		
	M	SD	M	SD	P
**Demographics**					
Age	64.6	8.5	67.0	9.4	0.40
Education	17.1	2.7	15.9	2.8	0.20
**Mini-Mental State Exam** 2	28.6	1.1	28.7	1.6	0.87
**Cognitive Speed/Flexibility** 3					
Trails A	48.4	9.9	46.1	7.5	0.33
Trials B	53.1	10.8	48.3	7.5	0.08
**Working Memory Span** 4					
Digits Forward/Backward	13.2	2.4	12.4	3.5	0.10
Spatial Span Forward	11.1	3.3	9.5	2.4	0.10
Spatial Span Backward	14.2	2.3	13.2	2.5	0.20
**Sustained Attention** 5					
Digit Vigilance Test	49.9	9.7	52.1	11.8	0.57
**Motor Speed** 6					
Finger Tapping (right hand)	50.0	8.4	45.0	10.2	0.41
**Structural MRI volumes** 7					
Bilateral putamen, GP	0.9	0.1	0.9	0.1	0.90
Bilateral caudate	0.5	0.1	0.5	0.1	0.10
Total Cortical Gray	28.1	1.4	27.0	2.6	0.09
Total Cerebellar Gray	6.2	0.8	6.3	0.8	0.78

1 Neuropsychological testing was conducted in PD participants when they were taking their medication.

2 The total score (maximum  =  30) is reported for the Mini-Mental State Exam [57].

3 T-scores are reported for the Trail Making Test [58].

4 Scaled scores are reported for the Digit Span and Spatial Span tests [59].

5 T-scores are reported for the Digit Vigilance Test [60].

6 T-scores for the dominant right hand are reported for the Finger Tapping Test [58].

7 Volumes are expressed as the percentage of estimated total intracranial volume (volume/eTIV).

Board-certified neurologists with a specialty in motor disorders assessed all PD participants (D.D. Song and S. Lessig). PD participants exhibited at least two of the three cardinal features of the disorder (i.e., bradykinesia, resting tremor, motor rigidity), were levodopa responsive, and did not exhibit features of progressive supranuclear palsy, corticobasal degeneration, multiple systems atrophy, or dementia. Eighteen PD participants were taking levodopa/carbidopa, and all were taking one or more DA agonists or releasers. The mean levodopa equivalence (LDE) was 748.3 (SD  =  428) [26]. Symptoms on the motor examination section of the United Parkinson's Disease Rating Scale (UPDRS) were worse off [Mean (SD)  =  29.6 (10.4)] than on medication [Mean (SD)  =  22.2 (8.0)] [F(1, 20) = 20.9, p<.0001, η^2^ = .51]. On the Hoehn and Yahr, 18 and 3 PD participants were stage 2 and 3, respectively, both off and on medication.

PD participants completed two fMRI sessions that were conducted on separate days at the same time of day. For one fMRI session, PD volunteers took their normal daily medication dosage one hour before scanning (ON condition) so that they were studied in an optimally medicated state. In the other fMRI session, participants refrained from taking medication for at least 2 half-lives of the longest acting medication or a minimum of 16 to 24 hours before the scan (OFF condition) so that they were studied in a ‘practical’ off state. The control subjects performed the fMRI task once in the scanner and once in the laboratory; only behavioral data from the scanning session were analyzed. For both groups, the order of testing conditions was counterbalanced. The study was approved by the University of California, San Diego (UCSD) Human Research Protections Program (HRPP). Study participants signed written informed-consent forms.

### Functional MRI

#### fMRI task

**Figure 1:**
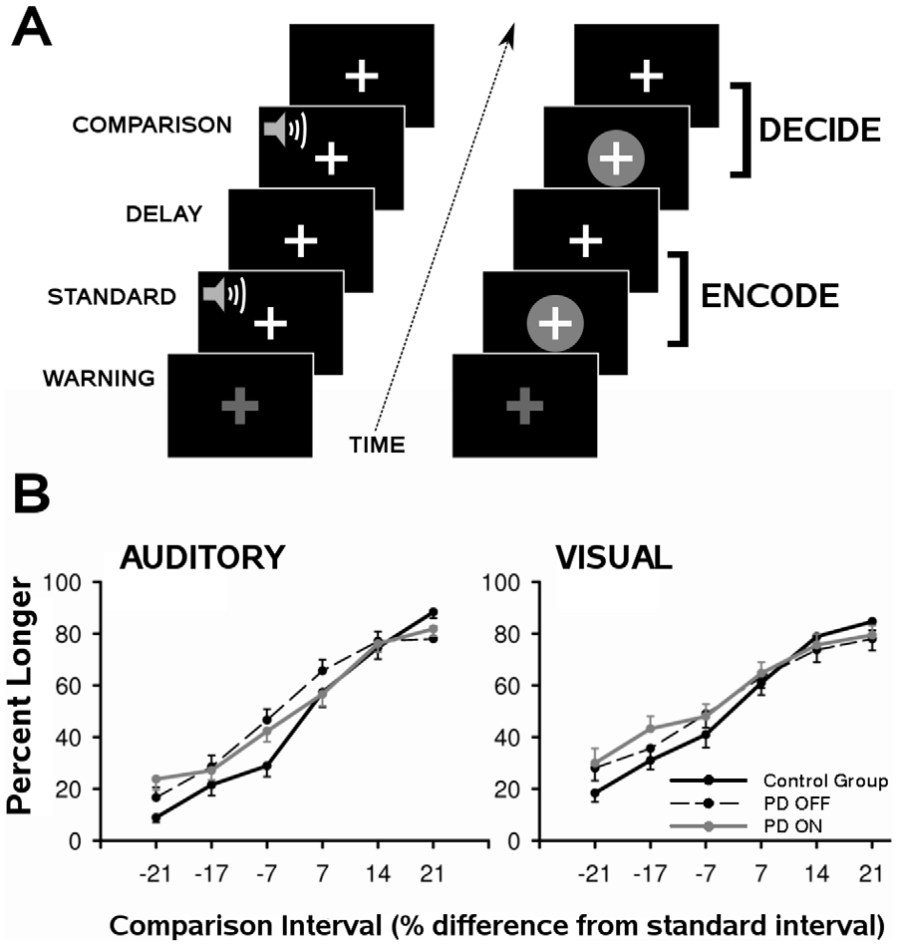
Time perception paradigm and task performance. A) Trial events for the auditory and visual conditions of the time perception task. Trials were preceded by a 500 ms warning signal (i.e., flashing yellow fixation cross and a mixed 700 Hz auditory tone). At trial onset, an auditory or visual standard-interval (SI) (1200 or 1800 ms) was presented and followed by a delay (6800 or 6200 ms). At 8 s post-trial onset, a comparison interval (CI) of the same modality was presented. Image acquisition (TR  =  2 s) was pegged to the onsets on the SI and CI. The first 12 s of a trial (i.e., equivalent to 6 TRs) constituted the *encoding phase*. The last 12 s of a trial constituted the *decision phase.* **B**) Mean (standard error bars) accuracy for the auditory (left) and visual (right) conditions in the control group and PD OFF and ON conditions. Accuracy data were converted to the mean percent longer, and averaged across the two SI conditions and their respective CIs. On the x axis, ±7, 14, and 21 designate CIs that were 7%, 14%, and 21% shorter (negative values) or longer (positive values) than the SI.

In the time perception task (Figure 1A), subjects attended to the duration of successively presented pairs of filled-auditory or visual stimuli, and then judged whether the second stimulus was shorter or longer than the first. Throughout the experiment, the subject maintained fixation on a white cross at the center of the display. One second before trial onset, a warning signal (i.e., flashing yellow cross and mixed 700-Hz tone) appeared for 500 ms followed by a 500 ms delay. Trial onset began with presentation of an auditory (1000 Hz pure tone) or visual (blue circle) SI that lasted 1200 or 1800 ms, and was respectively followed by a 6800 or 6200 ms delay. Then a CI of the same modality was presented, after which the subject indicated if it was shorter or longer than the SI by pressing a key with the right index or middle finger. For each SI, there were 3 shorter and 3 longer CIs that differed from the SI by successive increments of ±7%. Two SIs were used to help ensure that subjects encoded signal duration on each trial. The analyses collapsed across SI duration. The analyses also collapsed across signal modality, as there were no group differences in timing auditory and visual signals (see Results section), consistent with other reports [7]. Accuracy and reaction time (RT; from offset of the CI to key press) were measured. We did not include a sensorimotor control task since processing in sensory areas was of interest to our study.

There were 30 trials per SI condition (i.e., auditory 1200 ms, auditory 1800 ms, visual 1200 ms, and visual 1800 ms), with 5 trials per CI for a total of 120 trials. Trials were divided into 8 runs of 15 trials each. Within a run, SI conditions were randomly presented. Image acquisition was synchronized with the onset of the SI and the CI. Each trial included a minimum of 9 images (i.e., 18 s) to reduce the likelihood of nonlinear summing of overlapping hemodynamic responses. Additional one to five ‘filler’ 2.0 s epochs (fixation cross) were randomly added to the end of each trail (i.e., 45 filler images per run). Jitter in the inter-trial interval allowed for the best sampling of the hemodynamic response and establishment of a baseline resting state in the model (i.e., fixation plus ambient scanner noise). A run began and ended with 5 additional filler images to respectively allow for T1 equilibration and the delayed hemodynamic response of the final trial. Each run consisted of 190 images acquired over 6 min and 20 s.

#### Image acquisition

Imaging was conducted at the UCSD Center for FMRI using a GE 3-T Excite MRI system equipped with an 8-channel head-coil. Whole-brain blood-oxygen level dependent (BOLD) weighted echo-planar images were acquired using a single-shot, blipped, gradient-echo echo-planar pulse sequence (TE =  30 ms, TR =  2.0 s, 90° flip angle, FOV = 24 cm, resolution  =  64×64). Thirty-seven contiguous, axial 4-mm slices (3.75×3.75×4-mm voxel size) provided whole-brain coverage. High-resolution T1-weighted anatomic images were collected for anatomic localization (3D spoiled gradient-recalled at steady-state, TE = 3.0 ms, TR = 7.8 ms, 12° flip angle, number of excitations (NEX)  =  1, 1-mm slice thickness, FOV = 25 cm, resolution  =  256×256). Foam padding was used to limit head motion. Auditory stimuli were delivered binaurally through a headphone that together with earplugs attenuated background scanner noise by about 40 db. Visual stimuli were viewed through a mirror mounted on the head-coil.

#### Image analysis

Functional images were generated using Analysis of Functional NeuroImages (AFNI) software. Time series images were spatially registered in 3-dimensional space and corrected for time-slice acquisition differences. A deconvolution analysis (correcting for scanner drift) was used to generate impulse response functions (IRFs) of the fMRI signal on a voxelwise basis. Each IRF was estimated relative to the baseline state (i.e., filler images), without a priori assumptions about the shape, delay, or magnitude of the IRF. Six head-motion parameters were included as covariates of no interest. Estimates of percent signal change (PSC) for each image acquired 0 s to 24 s post-stimulus onset were then calculated by taking the beta coefficient and dividing it by the model intercept. The PSC maps were interpolated to volumes with 1-mm^3^ voxels, co-registered, converted to Talairach coordinate-space, and blurred using a 4-mm Gaussian root mean square filter.

#### Spatial extent analysis

This analysis examined the within-group spatial extent of activation. For each group (control, PD ON, PD OFF), statistical parametric maps were generated to identify voxels that exhibited a significant change in activation (i.e., PSC estimates) during each phase of the trial. Figure 1A shows that the first 12 s of a trial (i.e., 6 TRs) constituted the *encoding phase*, during which the subject encoded the SI and held it in memory; the last 12 s constituted the *decision phase*, wherein the subject encoded the CI and judged its duration relative to the SI. Repeated-measures ANOVAs for each group tested the effect of time (i.e., 6 TRs for each phase), separately for the encoding and decision phases of a trial. Voxelwise thresholds were derived from 3,000 Monte Carlo simulations (AFNI AlphaSim), which computed the voxel probability and minimum cluster-size threshold needed to obtain a .05 familywise alpha. Because spatial thresholds are biased against smaller activation clusters of a priori interest (i.e., basal ganglia and midbrain nuclei), statistical thresholds were derived separately for basal ganglia/midbrain and cortical volumes [27]. This was accomplished by creating a basal ganglia/midbrain mask (i.e., claustrum, putamen, globus pallidus (GP), caudate, substantia nigra (SN), red nucleus, and subthalamic nucleus) using the Talairach Daemon dataset; the mask was then expanded to include any voxels within a 2 mm radius. The cortical mask included all other regions of the brain including the cerebellum. Each mask was used in the Monte Carlo simulations to determine the appropriate combination of individual voxel-probability and minimum cluster-size threshold. For the basal ganglia/midbrain volume, a voxelwise threshold of p<.008 and a minimum cluster size of .225 ml yielded a .05 familywise alpha. For the cortical volume, a voxelwise threshold of p<.004 and a minimum cluster size of .338 ml yielded a .05 familywise alpha.

#### Functional region of interest analysis

A functional region of interest (fROI) analysis was conducted to evaluate potential group differences. A fROI map was generated by conjoining activated regions identified in the above spatial extent analyses across the control group and the PD ON and OFF conditions. Thus, any voxel significantly activated in at least one of the groups or conditions contributed to the final fROI map. Two conjunctive maps were generated, one for the encoding and one for the decision phase. Masks from the Talairach Daemon dataset were then used to separate large clusters into functionally relevant regions, which were the basis for all subsequent analyses.

Rather than focusing exclusively on peak activation, we analyzed group differences in the temporal dynamics or evolution of brain activity, since this might better characterize neuronal functioning as it does in normal aging [28]. This approach also avoided problems with assuming equivalence among regions in the time delay of hemodynamic responses to an event [29]. We first compared fROI between the control group and the OFF condition to evaluate the effects of disease on activation. The 2 (group; G) X 6 (time; T, where T_encoding phase_  =  the first 6 TRs and T_decision phase_  =  the last 6 TRs of a trial) mixed-model ANOVA tested for the main effect of group (G) and its interaction with time (G X T), separately for the encoding and the decision phases. Next, regions that showed significant group effects were compared between the OFF and ON conditions to evaluate medication effects. The 2 (medication condition; Med) X 6 (T) repeated-measures ANOVA tested the main effect of medication condition (Med) and its interaction with time (Med X T), separately for the encoding and decision phases. If medication had an effect, mixed-model ANOVAs compared the ON condition with the control group to determine if DA normalized activation. The significance threshold was set at p<.03 for the cortical volumes and at p<.05 for the basal ganglia/midbrain volumes as the latter involved far fewer comparisons. The Huynh-Feldt correction adjusted for heterogeneity of variance in multiple degrees-of-freedom tests.

#### Effective connectivity analysis

In addition to the univariate tests that assessed the effect of medication on activation of individual fROI, we also asked if DA therapy altered interactions of the striatum with the cortex and cerebellum. This was achieved by conducting voxel-based tests of psychophysiological interactions (PPI) separately for the encoding and decision phases [30]. Voxels in the bilateral caudate and putamen, which showed abnormal activation OFF medication, were the seed ROI and were selected for each subject using the conjunctive maps generated for the fROI analyses. The experimental variable was medication state (ON, OFF). Multiplication of the deconvolved time series for the seed areas with the experimental variable formed the interaction term (i.e., PPI regressor), which tested whether connectivity of the striatum with the whole brain was modulated by medication. A p<.005 voxelwise threshold and a .225 ml minimum cluster size was the criterion for significance.

### Structural MRI Analysis

To assess group differences in striatal, cerebral, and cerebellar volume, automated cortical reconstruction and volumetric segmentation of T1-weighted images was performed using FreeSurfer (http://surfer.nmr.mgh.harvard.edu/), which is a vertex-based approach. Processing included removal of non-brain tissue using a hybrid watershed/surface deformation procedure [31] and transformation to Talairach space. This was followed by segmentation of subcortical white- and deep gray-matter volumetric structures [32], intensity normalization [33], tessellation of the gray-white matter boundary, automated topology correction [34], [35], and surface deformation following intensity gradients to optimally place the gray/white and gray/cerebrospinal fluid borders at the location where the greatest shift in intensity defines the transition to the other tissue class [34]. Transformation and segmentations were manually verified. This approach provides anatomically accurate renderings of regional volumes, without potential rater bias [32]. To account for differences in head size, volumes were divided by estimated total intracranial volume (eTIV). Table 1 shows that striatum and cerebral/cerebellar gray-matter volumes did not differ between groups (p values >.05).

## Results

### fMRI Task Performance

Accuracy was converted to the percent longer responses for each CI, per convention. CIs that were increments of ±7%, 14% or 21% shorter/longer than the SI were averaged across the SI conditions. A mixed-model ANOVA compared the control group with the OFF condition, testing the main effect of group, modality (audition, vision), CI, and the interactions. The group X CI interaction [F(3.6,137.6) = 3.8, p<.005] was due to worse performance (i.e., flatter slope, lower accuracy) in the OFF condition than in the control group (Figure 1B). The modality X CI interaction [F(5,190) = 2.3, p<.05] indicated that performance was worse for visual than auditory intervals in both groups. No other significant effects were found. The OFF and ON conditions were then compared. The repeated-measures ANOVA showed no effect of medication on performance, irrespective of modality. This was consistent with a mixed-model ANOVA comparing the control group with the ON condition, which showed a group X CI interaction [F(3.2,121) = 3.4, p<.02]. No other group effects were found. As for RTs, group and medication effects were not significant (Mean (SE): Controls  =  2316 ms (68); OFF  =  2322 ms (65); ON  =  2404 ms (74)). However, RTs were longer for visual (Mean (SE)  =  2390 ms (54)) than auditory intervals (Mean (SE)  =  2292 ms (54) in both groups [F1,38) = 7.0, p<.01].

### fMRI Spatial Extent Analysis

**Figure 2:**
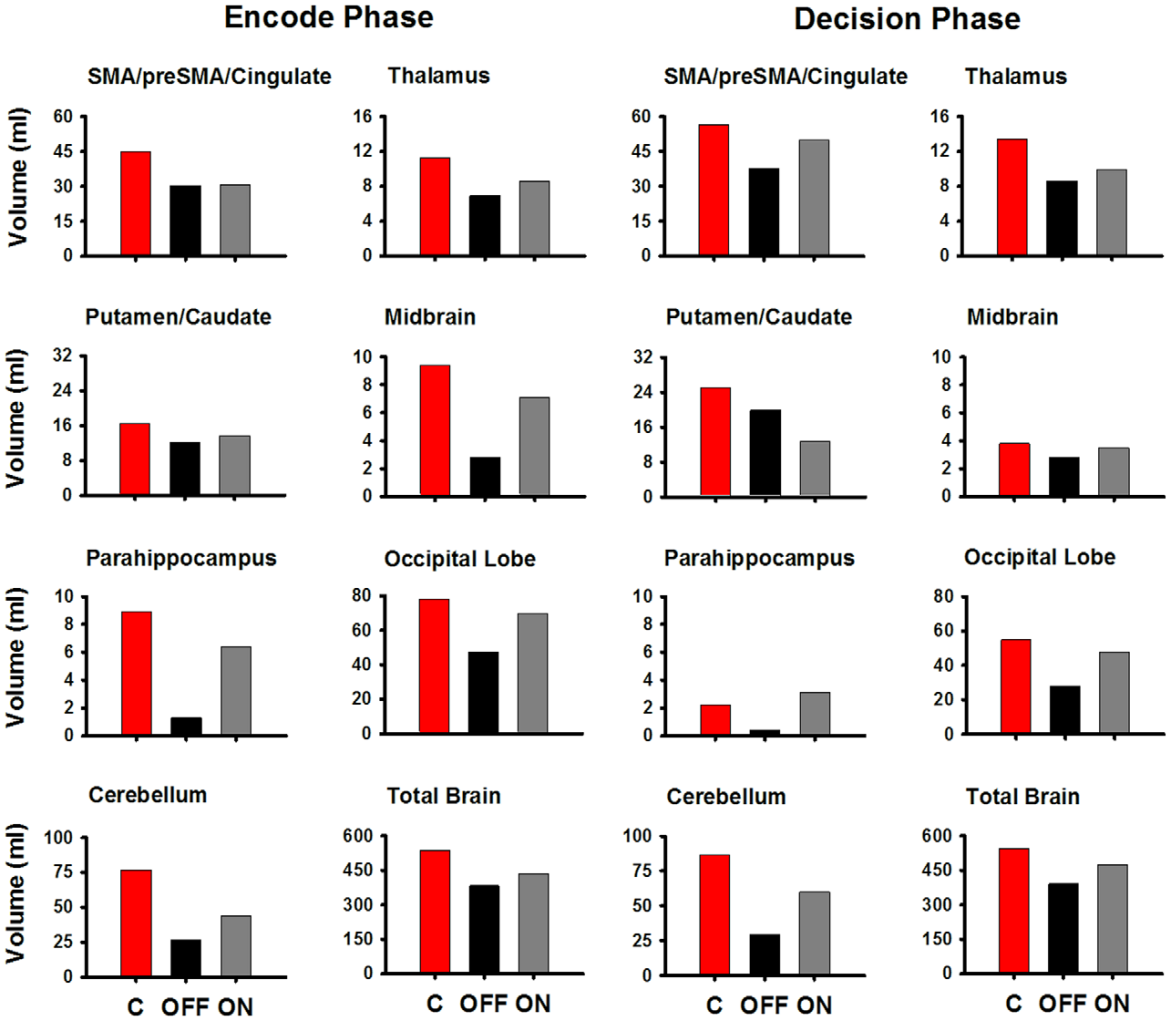
Spatial extent of activation in areas showing reduced volumes in the PD OFF condition. Activation volumes for the left and right hemispheres combined are displayed for the encoding and decision phases in the C (red bars), PD OFF (black bars) and PD ON (grey bars) groups. Areas are displayed that showed reduced volumes of more than 25 percent in the PD OFF condition relative to the control (C) group. See Figures S1 and S2 for activation volumes in the whole brain, and Tables S1 and S2 for details about activation foci.

Descriptive analyses of the spatial extent of activation indicated that total volume of activation was reduced in both phases by approximately 28% in the PD OFF group relative to the controls. Frontal, parietal, and temporal lobe volumes were reduced 15% to 20% in the OFF group, irrespective of phase. However, Figure 2 shows that for both phases volume reductions of more than 25% were seen OFF medication in the bilateral preSMA/SMA/cingulate (33% for both phases), thalamus (36% to 39%), striatum (27% to 30%), midbrain nuclei (27% to 30%), parahippocampus (83% to 85%), occipital lobe (39% to 49%), and cerebellum (65% for both phases). Whereas medication appeared to increase activation extent, Figure 2 shows that the spatial extent of activation typically remained lower relative to the control group (see Figures S1 and S2 for activation volumes in the whole brain, and Tables S1 and S2 for details about activation foci).

### Functional ROI Analysis

**Figure 3:**
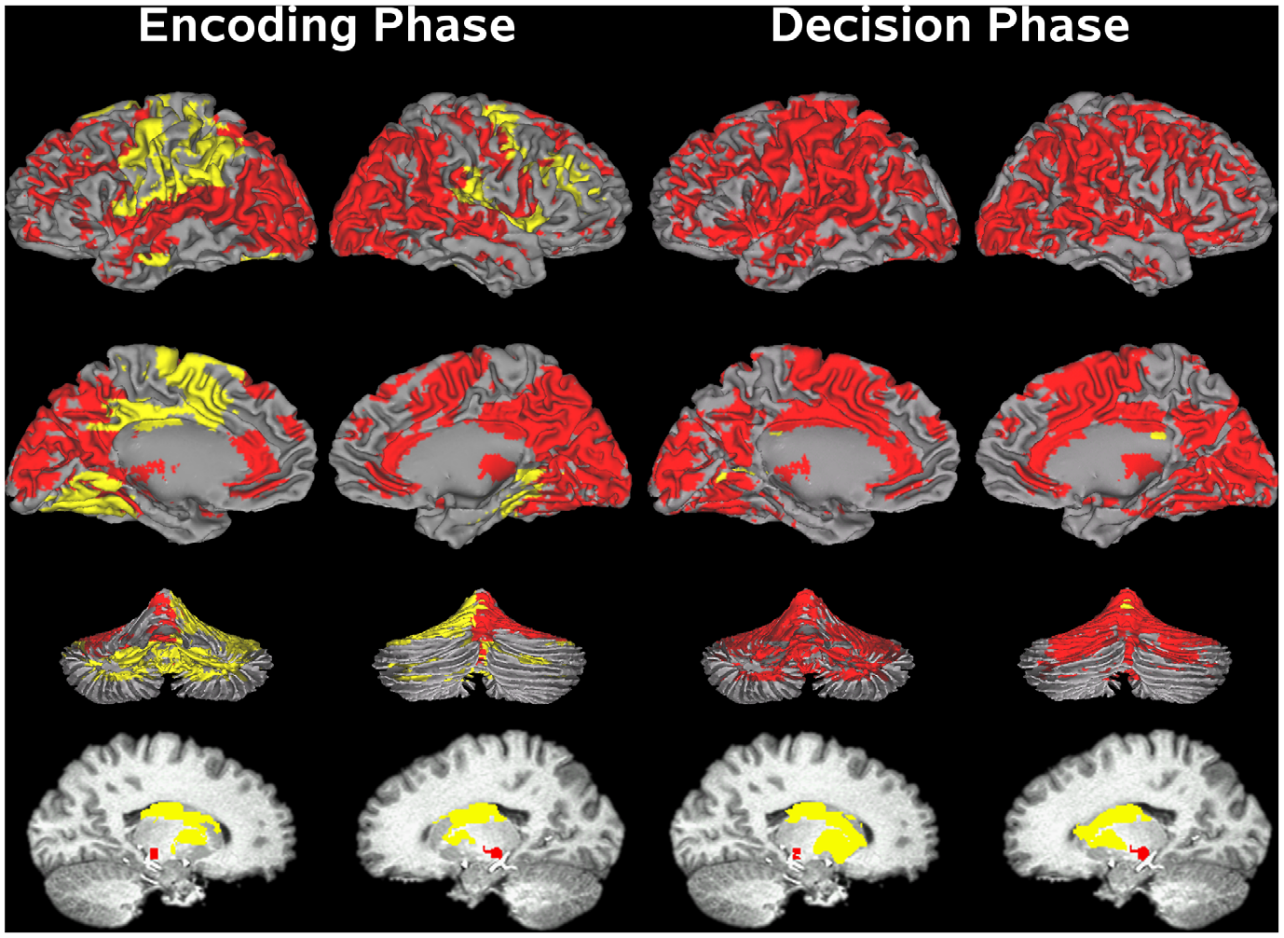
Functional ROI for the encoding (A) and decision phases (B). Functional ROIs were derived from conjoining activation maps in Figures S1 and S2, respectively. Yellow regions designate significant group (Control vs. PD OFF) differences in activation; red regions indicate no significant group differences. Brain activation is projected onto the lateral and medial surfaces of the left and right hemispheres (rows 1 and 2), the anterior and posterior surfaces of the cerebellum (row 3), and the left and right basal ganglia (row 4). See Table S3 for details about individual fROI.

The conjoined fMRI activation-masks in Figure 3 show that similar regions of activation were found during both phases of the trial (see Table S3 for details about activation foci). Figure 3 also shows that group differences were found in only a subset of these regions (i.e., yellow), and were partially related to the behavioral context. For example, the PD OFF group exhibited abnormal preSMA/SMA/cingulate, precentral, middle-frontal, parietal, insula, inferior-temporal, right-parahippocampus, and lateral-cerebellum activation during the encoding phase. In contrast, posterior-cingulate and left-parahippocampus activations were abnormal during the decision phase. Only the striatum and vermis exhibited abnormal activation during both phases. We now turn to the statistical analyses in support of these observations.

#### Encoding phase: PD OFF versus control

**Figure 4:**
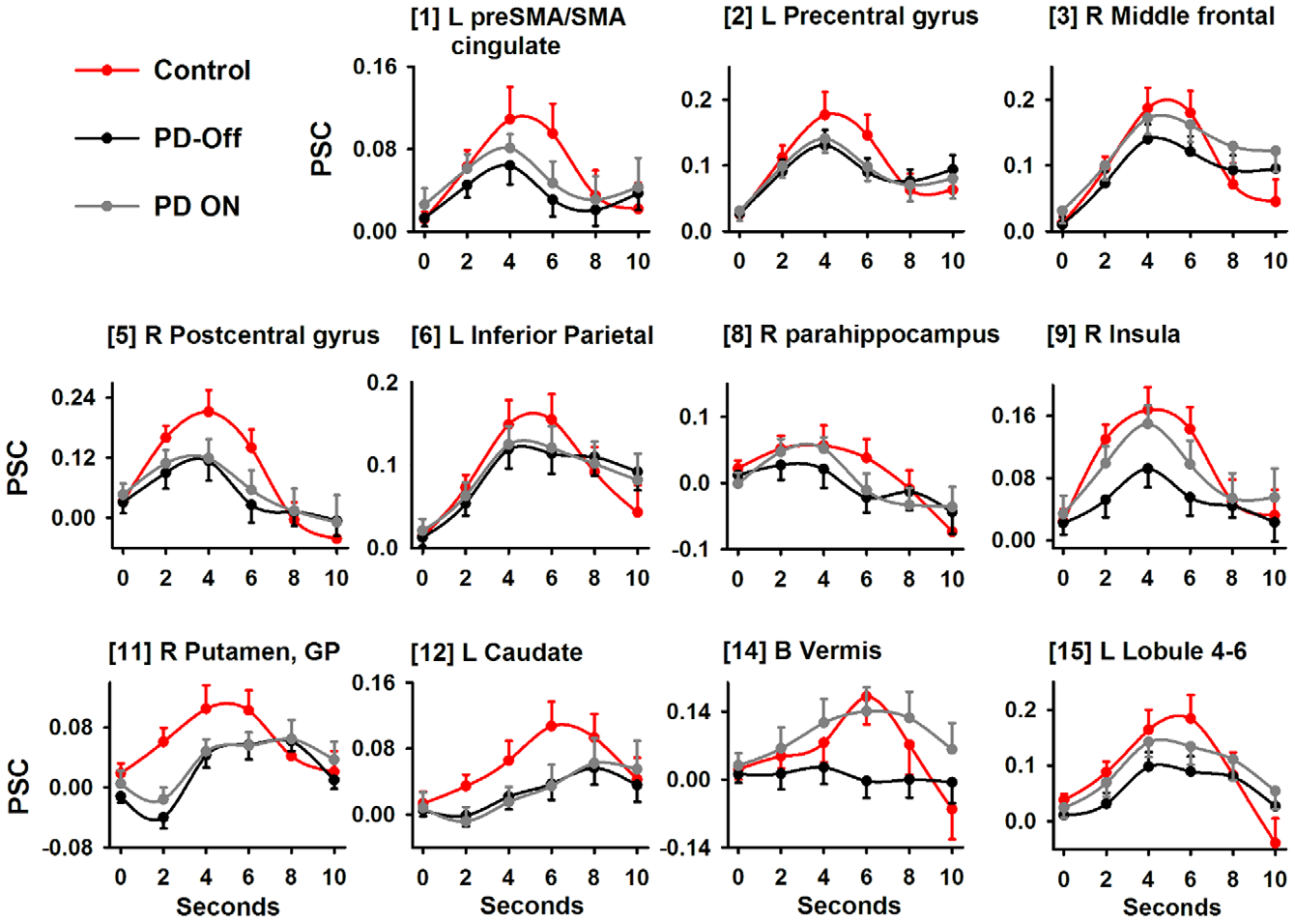
Percent signal change in regions showing abnormal activation OFF medication during the encoding phase. Graphs display representative regions showing different time courses of activation between the control (red lines) and PD OFF (black lines) groups (G X T). The effect of medication is also shown (PD ON; gray lines). The abscissa designates the time (sec) post-trial onset. The mean (standard error bars) percent signal change (PSC) is graphed for the first half of the hemodynamic response (i.e., encoding period). Bracketed numbers reference regions detailed in Table 2. B  =  bilateral hemispheres; L  =  left hemisphere; R  =  right hemisphere; SMA  =  supplementary motor area.

Table 2 lists regions wherein activation during interval encoding differed between the control and PD OFF groups, and Figure 4 displays activation time-courses in representative regions for the first half of the hemodynamic response (i.e., encoding period). This figure shows that hemodynamic responses return to baseline in some fROI, but not in others because the trial extends beyond the encoding of the SI, including activity related to WM maintenance. In all regions, the temporal dynamics of activation differed between the groups (G X T). In the striatum, hemodynamic responses were attenuated and temporally lagged OFF medication relative to controls. Hemodynamic responses were also attenuated OFF medication and returned to baseline levels soon after peak activation in left preSMA/SMA/cingulate, left precentral gyrus, bilateral postcentral gyrus, left inferior-temporal cortex, and right insula; by comparison, activation in the control group was robust for longer periods. In contrast, right middle-frontal gyrus (MFG), left inferior-parietal cortex, and bilateral lateral cerebellar activity was attenuated OFF medication and prolonged relative to the control group. Moreover, no significant activation was seen OFF medication in the right parahippocampus and vermis.

**Table 2:** Regions showing significant group and medication effects during the encoding phase.

						Control vs PD OFF 1	PD OFF vs PD ON 2
Region	BA	X	Y	Z	ml	Group X Time	Medication X Time
**Frontal**							
[1] L preSMA/SMA, cingulate	6,24,31	−8	−7	45	24.1	.02	
[2] L Precentral	4,6	−43	−7	37	17.5	.03	
[3] R Middle	6,9,10,46	36	19	37	17.7	.01	
**Parietal**							
[4] L Postcentral	2,3	−45	−23	37	9.0	.005	
[5] R Postcentral	3,5	56	−18	22	3.5	.01	
[6] L Inferior	40	−46	−41	37	19.0	.03	
**Temporal**							
[7] L Inferior	20	−57	−15	−19	9.6	.025	
[8] R parahippocampus	36	25	−36	−7	6.0	.03	.01
[9] R Insula (anterior & posterior)	13	40	−8	9	12.0	.025	.02
**Basal Ganglia**							
[10] L Putamen, GP		−24	−3	5	7.1	.01	
[11] R Putamen, GP		25	−4	5	6.7	.005	
[12] L Caudate (body, tail)		−20	−13	15	4.5	.05	
[13] R Caudate (body, tail)		21	−16	16	3.5	.05	
**Cerebellum**							
[14] B Vermis		0	−63	−3	4.8	.006	.03
[15] L Lobule 4–6		−19	−56	−16	2.5	.008	
[16] L Lobule 7–10		−21	−60	−34	20.8	.025	
[17] R Lobule 7–10		23	−59	−34	19.3	.02	

fROIs are displayed in Figure 3 (left column; yellow). Brodmann areas (BA) were defined by the Talairach and Tournoux atlas. Cerebellar lobules were defined by the Schmahmann atlas [61]. Coordinates represent distance in mm from anterior commissure: x, right(+)/left (−); y, anterior (+)/posterior (−); z, superior (+)/inferior (−). B  =  bilateral hemispheres; L =  left hemisphere; R  =  right hemisphere; GP  =  globus pallidus; SMA  =  supplementary motor area.

1 This column summarizes areas showing significant differences between the control and PD OFF groups in the temporal dynamics of activation during the first half of the hemodynamic response (encoding period; G X T).

2 This column summarizes areas showing significant differences between the ON and OFF medication conditions in the temporal dynamics of activation (Med X T).

#### Encoding phase: Medication

Table 2 and Figure 4 show that medication altered activation in the right parahippocampus, the right insula and the vermis (Med X T). In all three regions, comparisons between the ON and the control groups indicated that medication normalized activation.

#### Decision phase: PD OFF versus control

**Figure 5:**
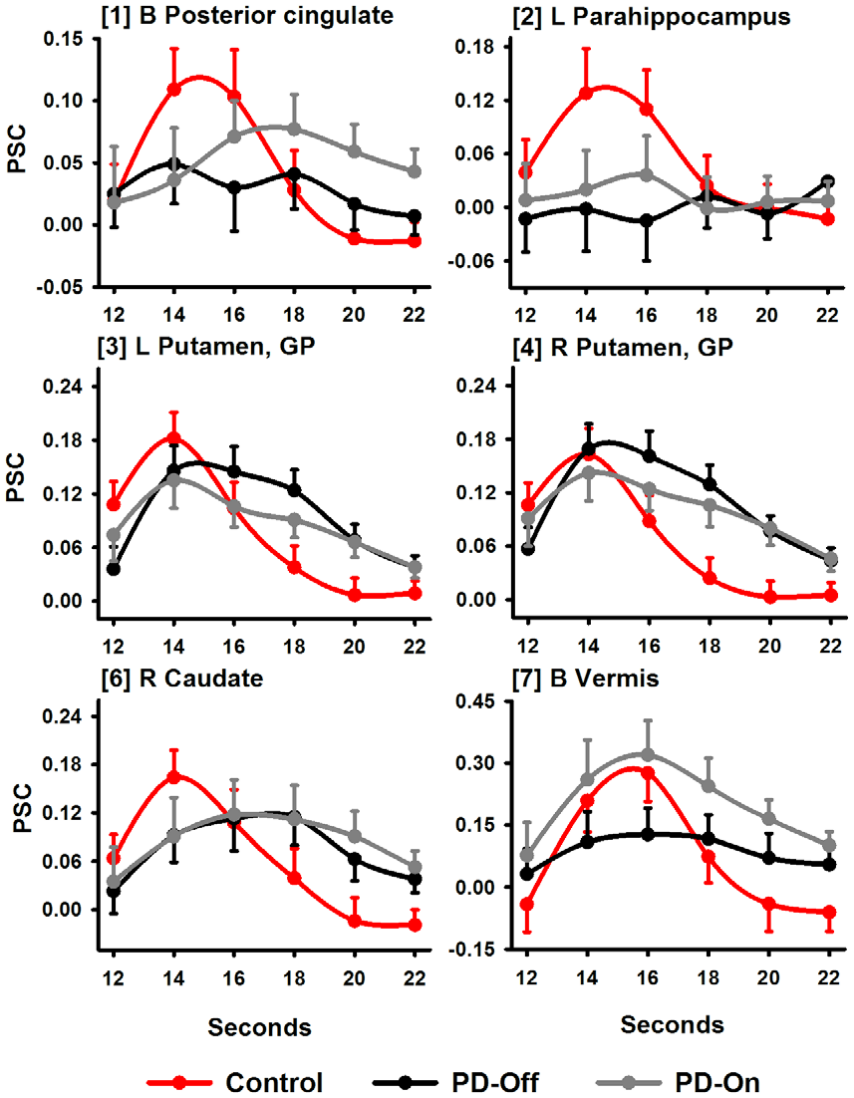
Percent signal change in regions showing abnormal activation OFF medication during the decision phase. Graphs display regions showing different time courses of activation between the control (red lines) and PD OFF (black lines) groups (G X T) during the decision phase. The effect of medication is also shown (PD ON; gray lines). The abscissa designates the time (sec) post-trial onset. The mean (standard error bars) percent signal change (PSC) is graphed for the second half of the hemodynamic response (i.e., decision phase). Bracketed numbers reference regions detailed in Table 3. B  =  bilateral hemispheres; L  =  left hemisphere; R  =  right hemisphere; GP  =  globus pallidus.

Table 3 and Figure 5 show regions wherein the temporal dynamics of activation during decision making differed between the controls and the OFF condition (G X T). In bilateral putamen, peak activation was normal, but prolonged relative to controls. In bilateral caudate, peak activation was lagged, attenuated and prolonged relative to controls. No significant activation was seen in the posterior cingulate and the left parahippocampus, and there was only a trend (p<.05) for vermis activation; these results contrasted with significant activation in the control group in all three regions. No other group differences were found.

**Table 3:** Regions showing significant group and medication effects during the decision phase.

						Control vs. PD OFF 1	PD OFF vs. PD ON 2
Region	BA	X	Y	Z	ml	Group X Time	Medication X Time
** Parietal**							
[1] B Posterior cingulate	23	1	−33	24	.5	.03	
** Temporal**							
[2] L parahippocampus	36	−19	−46	3	.5	.01	
** Basal Ganglia**							
[3] L Putamen, GP		−23	−2	3	9.8	.004	.03
[4] R Putamen, GP		24	−2	5	7.8	.003	.03
[5] L Caudate (head, body, tail)		−15	−4	13	6.4	.025	
[6] R Caudate (head, body, tail)		13	2	15	5.1	.003	
** Cerebellum**							
[7] B Vermis (anterior)		0	−63	−3	.6	.01	.05

fROIs are displayed in Figure 3 (right column; yellow). Brodmann areas (BA) were defined by the Talairach and Tournoux atlas. Coordinates represent distance in mm from anterior commissure: x, right(+)/left (−); y, anterior (+)/posterior (−); z, superior (+)/inferior (−). B  =  bilateral hemispheres; L =  left hemisphere; R  =  right hemisphere; GP  =  globus pallidus.

1 This column designates areas wherein the temporal dynamics of activation during the second half of the hemodynamic response (decision period) differed between the control and PD OFF groups (G X T).

2 This column designates areas wherein the temporal dynamics of activation differed between the OFF and ON medication conditions (Med X T).

#### Decision phase: Medication

Table 3 and Figure 5 show that medication altered activation time-courses only in the bilateral putamen and vermis. Medication significantly attenuated the prolonged time-courses of putamen activity, but did not normalize activation relative to the control group (G X T: p<.025). In contrast, medication enhanced and normalized vermis activity.

### Effective Connectivity Analysis

**Figure 6:**
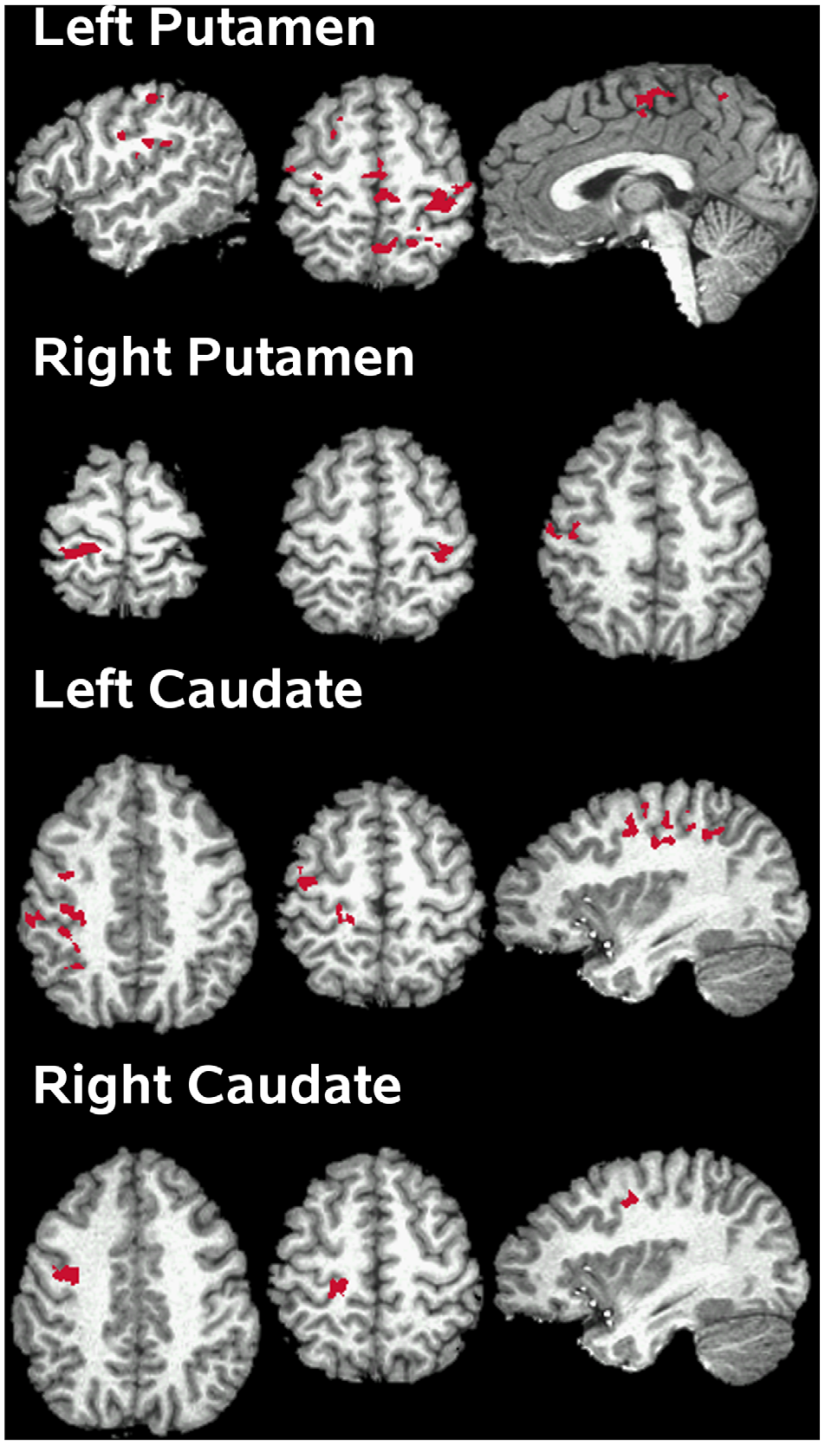
Cortical regions showing connectivity with the striatum that was modulated by medication. For each striatal seed region, activation is projected onto the lateral and medial surfaces of the left and right hemispheres, which are displayed in neurological view. See Table 4 for details about individual activation foci.

**Figure 7:**
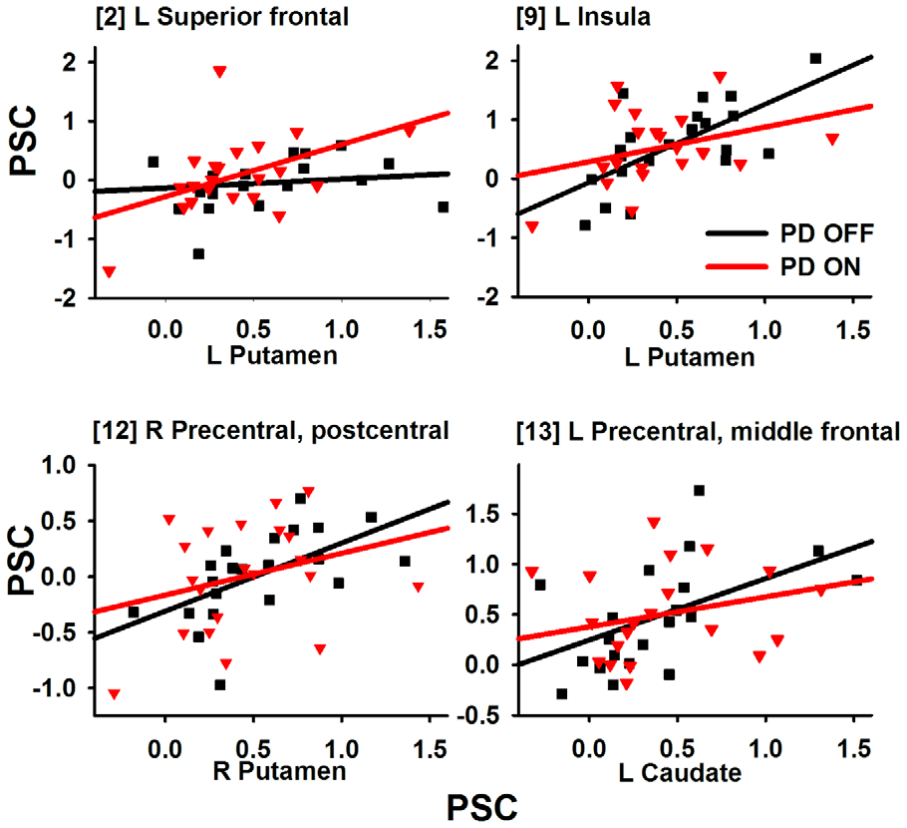
Scatter plots showing significant striatal-cortical connectivity that was modulated by medication. Scatter plots show the relationship between striatal and cortical activity as a function of medication state in representative regions. Linear regression fits are overlaid on the scatter plots. Gray squares and lines  =  PD OFF condition; black triangles and lines  =  PD On condition. Bracketed numbers refer to regions listed in Table 4.

Effective connectivity analyses showed that interactions of the striatum with the cortex were modulated by DA therapy, but only in the decision phase. Table 4 and Figure 6 show that DA therapy significantly modulated interactions between the striatum and the cortex (i.e., SMA, precentral and postcentral gyrus, superior frontal gyrus (SFG), MFG, superior and inferior parietal cortex, precuneus, and insula), but not the cerebellum. Figure 7 graphs representative PPI. Corticostriatal interactions were stronger OFF than ON medication. An exception was stronger connectivity ON than OFF therapy between the left putamen and the left SFG (BA 6).

**Table 4:** Cortical regions showing effective connectivity with the striatum that was modulated by medication.

Basal Ganglia Seed – Interacting Regions	BA	X	Y	Z	ml
** Left Putamen**					
[1] B SMA	6	2	−19	55	1.0
[2] L Superior frontal 1	6	−22	7	57	.2
[3] L Precentral gyrus	6	−46	−12	28	.2
[4] L Precentral, postcentral gyrus	3,4	−23	−32	62	.6
[5] L Precentral, postcentral, inferior parietal	3, 4, 40	−37	−24	45	2.3
[6] R Precentral, postcentral, superior parietal	3, 4, 7	29	−36	56	2.0
[7] L Inferior parietal	40	−47	−30	25	.4
[8] B Precuneus	7	5	−48	53	.3
[9] L Insula	13	−49	−26	19	.5
** Right Putamen**					
[10] L Precentral gyrus	4	−44	−16	49	.2
[11] L Precentral, postcentral gyrus	3,4	−22	−34	64	.6
[12] R Precentral, postcentral gyrus	3, 4	31	−28	56	.3
** Left Caudate**					
[13] L Precentral, middle frontal	6	−37	−9	48	1.0
[14] L Precentral	4	−35	−21	37	.3
[15] L Precentral, postcentral gyrus	3,4	−18	−31	54	.4
[16] L Postcentral gyrus, inferior parietal	2, 3, 40	−42	−30	44	1.0
[17] L Inferior parietal	40	−30	−42	43	.7
** Right Caudate**					
[18] L Middle frontal	6	−34	−6	41	.3
[19] L Precentral, postcentral gyrus	3,4	−21	−32	57	.7

Regions showing effective connectivity each striatal seed are displayed in Figure 6. Brodmann areas were defined by the Talairach and Tournoux atlas. Coordinates represent distance in mm from anterior commissure: x, right(+)/left (−); y, anterior (+)/posterior (−); z, superior (+)/inferior (−). B  =  bilateral hemispheres; L  =  left hemisphere; R  =  right hemisphere; SMA  =  supramarginal gyrus.

1 The correlation between a basal ganglia seed and a cortical region was typically greater OFF than ON medication. An exception was the correlation between the left putamen and the left superior/middle frontal cortex (BA 6), which was greater ON than OFF medication.

## Discussion

The present study uncovered four new findings that elucidated neurobehavioral mechanisms of time perception deficits in PD and their response to DA therapy. First, impaired time perception OFF medication was associated with abnormal activation in systems regularly associated with time perception in studies of healthy adults [4], [20], [36]–[38] including the striatum, selected cortical sites (e.g., preSMA/SMA, cingulate gyrus, precentral and postcentral gyri, insula, middle-frontal and inferior-parietal cortex, parahippocampus) and the cerebellum (lobules, vermis). Unlike studies of timed movement, our results could not be attributed to classic motor-control functions of the basal ganglia or the cerebellum. Second, for the first time we were able to better distinguish temporal and non-temporal sources of cognitive dysfunction in time perception by separating activity during the encoding and decision phases. Our finding of striatal dysfunction in both phases was consistent with the role of DA neurotransmission in timekeeping operations. In addition, our results suggested that non-temporal operations emphasized by each phase were also disrupted in PD. Specifically, activation was abnormal in a classic WM network (middle frontal-inferior parietal, SMA, lateral cerebellum) during the encoding phase, whereas activation was absent in a network that mediates executive processes and memory (posterior-cingulate, parahippocampus) during the decision phase. Third, another novel finding was that neuronal dysfunction in PD was sometimes characterized by abnormal temporal dynamics. For example, hemodynamic responses of the striatum were typically attenuated, but also temporally lagged and sometimes prolonged; activity was also prolonged in a WM network (middle frontal-inferior parietal cortex, lateral cerebellar). These findings cannot be explained by longer RTs in PD, which did not differ from the control group. Fourth, DA therapy did not alleviate time perception deficits, despite its robust benefit on motor symptoms (UPDRS). Our fMRI results suggested that this was likely due to insufficient restoration of neuronal activation and perhaps corticostriatal effective-connectivity. We now discuss these findings more completely.

### Striatal Dysfunction in PD

As predicted, striatal activation OFF medication was abnormal during both phases of a trial, consistent with its role in timekeeping. During interval encoding, attenuated and temporally lagged striatal activity may suggest weakened and delayed processing of input from multiple corticostriatal loops. Theoretically, weakened and lagged striatal activation should disrupt integration of cortical input over time, a key function of the ‘core timer.’ Specifically, by the striatal beat frequency (SBF) model, sensory input is translated into cortical oscillatory patterns, which support the clock signal, and striatal medium-spiny neurons serve as a core timer by detecting and integrating oscillatory states over time [2]. Thus, impaired time perception in PD may be partially related to deficient striatal integration of cortical input. This may be due to diminished nigrostriatal phasic-DA, which it thought to signal the onset and offset of a to-be-timed event [2].

We also found striatal dysfunction during the decision phase, wherein peak activation of the putamen/GP was normal in PD, yet sustained longer relative to the control group; caudate activity was also sustained, but temporally lagged and attenuated. Although both phases involve timekeeping, the duration of two intervals is compared only during the decision phase. Sustained striatal activity leads us to speculate that it reflects compensation for difficulties in additional processes including updating WM with the CI [39] and comparing it with the SI. These processes require fast, flexible striatal-reactions, which are our study shows are temporally lagged and sometimes prolonged in PD during decision making and may be a basis for cognitive inflexibility [25].

### Disease-Related Cortical and Cerebellar Activity

Time perception impairments OFF medication were also associated with abnormal cortical and cerebellar activity. A unique finding was that these abnormalities partially depended on the context, despite activation of similar regions in both phases (Figure 3 and Table S3 for details about activation foci for each phase). During the encoding phase, hypoactivation was found in the cerebellum (lobules, vermis) and cortical areas of the motor (preSMA/SMA/cingulate, precentral/postcentral gyrus), executive (middle-frontal, inferior parietal), and limbic (parahippocampus, insula, inferior temporal) corticostriatal loops [40]. In the motor loop, preSMA/SMA dysfunction only during encoding was notable as it is at odds with a fundamental role for the SMA in timekeeping [22]. SMA dysfunction is common in PD and is traditionally attributed to difficulties with internally-generated behaviors. However, a more pivotal distinction may relate to its role in WM, specifically online storage of output from the motor pathway [4], [41]. By this proposal, SMA dysfunction in PD should be manifested particularly when maintenance is emphasized, as during the encoding phase wherein the SI is held in memory for over 6 s.

Memory-related difficulties during interval encoding were also suggested by PD participants' prolonged activation in a classic WM-network (middle-frontal, precentral, inferior-parietal, lateral cerebellum) [42]. This may suggest ‘compensatory processing’ for diminished preSMA/SMA functioning, but perhaps also for hypoactivity of the insular cortex, which has efferent connections to WM networks and the striatum [43]. The insula integrates processing from disparate domains (e.g., interoception, emotion, WM) including time [4], [36], [37], [44], [45]. Recent models suggest that the insula is an attentional ‘hub’ that assists central executive networks in generating accurate responses to salient or task-relevant events [46], [47]. In our study, insula dysfunction may be manifested during interval encoding due to uncertainty about the to-be-attended modality at trial onset, which accentuates attentional demands. This prospect is compatible with normal insula activation during the decision phase, wherein top-down attention mechanisms might enhance insula activation since the CI modality is certain as it always matches the SI. Memory-related deficits OFF therapy were further evidenced in the encoding phase by the striking absence of activity in the right parahippocampus, damage to which disrupts time perception [48].

Cortical dysfunction during the decision phase was unexpectedly confined to a medial ‘default-mode network’ (left parahippocampus, bilateral posterior cingulate) [49]. Activation was absent in this network OFF medication, in contrast to the control group. To our knowledge, parahippocampus dysfunction has not been reported in non-demented PD, whereas evidence for posterior-cingulate dysfunction remains debated [50]–[52]. We speculate that abnormal functioning of this network emerged during decision making due to the emphasis on executive processes (e.g., comparison of the SI and CI) and/or retrieval, which are modulated by this system [49]. Together with the absence of activity in the right parahippocampus during the encoding phase, deficient functioning of this network may underlie distortions in memory for time in PD [9], and may be an early marker of memory problems.

It was notable that vermis activity was greatly reduced OFF medication during both phases, as was lateral cerebellar activity during encoding. Our results contrast with cerebellar hyperactivity in PD during timed movements [17]–[19], which is attributed to compensation for striatal dysfunction. Although direct support for this proposal is lacking, hyperactivity of the cerebellum during timed movement, but not time perception may reflect an increased reliance on sensorimotor-coordination functions of the cerebellum, rather than purported timekeeping functions [53].

### Dopamine Modulation of Brain Activation and Corticostriatal Connectivity

Our results showed that DA therapy did not alleviate time perception deficits, consistent with some [14], but not all studies [9], [11], [12]. At first glance our results seem to conflict with the effects of DA agonists and antagonists on timing in animals [1]. However, our fMRI findings suggest that time perception does not benefit from DA therapy when activity is not sufficiently reinstated in timekeeping and non-temporal control systems known to mediate time perception. Specifically, during the encoding phase, treatment did not alter activation in the striatum or a WM network (SMA, middle frontal-inferior parietal cortex, lateral cerebellum), nor did it affect interactions of the striatum with other brain areas (effective connectivity). Likewise, during the decision phase impoverished activity in the default mode network (posterior cingulate, left parahippocampus) was not improved by DA therapy, nor was activity in the caudate, which interacts with this network [54].

There were some benefits of medication on brain activation, but they were circumscribed. During the encoding phase, treatment normalized attenuated limbic system (right insula and right parahippocampus) and vermis activity, possibly via the mesocortical-DA system. During the decision phase, DA therapy normalized vermis activity and improved, but did not restore the temporal dynamics of the putamen. Interestingly, DA therapy also mediated striatal interactions with the cortex, but not the cerebellum. Two patterns of effective connectivity were found, consistent with a recent study of motor timing in PD [19]. First, the striatum showed greater connectivity OFF than ON medication with the MFG, SMA, precentral/postcentral cortex, insula, and parietal cortex. Enhanced connectivity OFF therapy might appear counterintuitive since corticostriatal connectivity can be reduced OFF medication relative to controls [55]. However, animal models suggest that enhanced connectivity after DA depletion may reflect excessive synchronicity in corticostriatal circuits [56], which disrupts rapid, flexible updating/integration by the striatum in contexts that call for cognitive flexibility [25], such as the decision phase. Second, we also found that the putamen showed greater connectivity ON than OFF medication with the left SFG. Enhanced connectivity ON medication could reflect reduced inhibitory output from the striatum to an area that normally supports temporal decision-making [4], [20]. These speculations require further research as mechanisms of effective-connectivity in PD and their regulation by DA are not understood. Altogether, our finding of DA-mediated striatal activation and connectivity only during the decision phase is consistent with its effects on flexibility control-mechanisms, which are driven by the striatum and nigrostriatal DA [25]. Nevertheless, functioning is not restored in networks important for time perception.

### Summary

The present results illuminated the neurobehavioral mechanisms of time perception deficits in PD by distinguishing abnormal activity in the encoding and decision phases of a trial, which we hypothesized would both engage timekeeping, but accentuate demands on different non-temporal control processes. As predicted, neuronal dysfunction was found in a purported timekeeping center (striatum) in both phases. In addition, neuronal dysfunction in the encoding and decision phases was respectively manifested in WM/attentional (SMA, middle-frontal and inferior-parietal cortex, lateral cerebellum, insula, right parahippocampus) and executive processing/memory (left parahippocampus, bilateral posterior cingulate) centers. The temporal dynamics of activation in PD were also abnormal in the striatum and a frontoparietal-cerebellar network, which better characterized the basis for disturbances in timekeeping (temporally lagged and/or prolonged) and WM (prolonged). However, DA therapy did not alleviate time perception deficits in PD. Our fMRI results suggested that restoration of neuronal functioning was insufficient, possibly due to largely tonic effects of treatment, which fail to restore the balance of phasic and tonic DA in nigrostriatal and mesocortical pathways [8].

## Supporting Information

Figure S1 Regions (red) showing significant task-related activation during the encoding phase in analyses conducted separately for each of the three groups. Brain activation is projected onto the lateral and medial surfaces of the left (rows 1 and 2) and right hemispheres (rows 3 and 4), the anterior (row 5) and posterior (row 6) surfaces of the cerebellum, and sagittal sections of the left (row 7) and right (row 8) basal ganglia. See Table S1 for details about individual activation foci.
(DOC)

Figure S2 Regions (red) showing significant task-related activation during the decision phase in analyses conducted separately for each of the three groups. Brain activation is projected onto the lateral and medial surfaces of the left (rows 1 and 2) and right hemispheres (rows 3 and 4), the anterior (row 5) and posterior (row 6) surfaces of the cerebellum, and the left (row 7) and right (row 8) basal ganglia. See Table S2 for details about individual activation foci.
(DOC)

Table S1 Activation foci for each group during the encoding phase of a trial.
(DOC)

Table S2 Activation foci for each group during the decision phase of a trial.
(DOC)

Table S3 Functional ROI resulting from the conjunction of activations in the Control, PD OFF, and PD On Groups.
(DOC)

## References

[1] Meck WH (1996). Neuropharmacology of timing and time perception.. Cogn Brain Res.

[2] Matell MS, Meck WH (2004). Cortico-striatal circuits and interval timing: coincidence detection of oscillatory processes.. Cogn Brain Res.

[3] Gibbon J, Church RM, Meck WH (1984). Scalar timing in memory.. Ann NY Acad Sci.

[4] Harrington DL, Zimbelman JL, Hinton SC, Rao SM (2010). Neural modulation of temporal encoding, maintenance, and decision processes.. Cereb Cortex.

[5] Harrington DL, Haaland KY, Hermanowicz N (1998). Temporal processing in the basal ganglia.. Neuropsychol.

[6] Perbal S, Deweer B, Pillon B, Vidailhet M, Dubois B (2005). Effects of internal clock and memory disorders on duration reproductions and duration productions in patients with Parkinson's disease.. Brain Cogn.

[7] Smith JG, Harper DN, Gittings D, Abernethy D (2007). The effect of Parkinson's disease on time estimation as a function of stimulus duration range and modality.. Brain Cogn.

[8] Rammsayer TH (1997). Are there dissociable roles of the mesostriatal and mesolimbocortical dopamine systems on temporal information processing in humans?. Neuropsychobiology.

[9] Malapani C, Deweer B, Gibbon J (2002). Separating storage from retrieval dysfunction of temporal memory in Parkinson's disease.. J Cogn Neurosci.

[10] Jones CR, Malone TJ, Dirnberger G, Edwards M, Jahanshahi M (2008). Basal ganglia, dopamine and temporal processing: Performance on three timing tasks on and off medication in Parkinson's disease.. Brain Cogn.

[11] Merchant H, Luciana M, Hooper C, Majestic S, Tuite P (2008). Interval timing and Parkinson's disease: heterogeneity in temporal performance.. Exp Brain Res.

[12] Pastor MA, Artieda J, Jahanshahi M, Obeso JA (1992). Time estimation and reproduction is abnormal in Parkinson's disease.. Brain.

[13] McIntosh GC, Brown SH, Rice RR, Thaut MH (1997). Rhythmic auditory-motor facilitation of gait patterns in patients with Parkinson's disease.. J Neurol Neurosurg Psychiatry.

[14] Guehl D, Burbaud P, Lorenzi C, Ramos C, Bioulac B (2008). Auditory temporal processing in Parkinson's disease.. Neuropsychologia.

[15] Koch G, Costa A, Brusa L, Peppe A, Gatto I (2008). Impaired reproduction of second but not millisecond time intervals in Parkinson's disease.. Neuropsychologia.

[16] Elsinger CL, Rao SM, Zimbelman JL, Reynolds NC, Blindauer KA (2003). Neural basis for impaired time reproduction in Parkinson's disease: an fMRI study.. J Int Neuropsychol Soc.

[17] Cerasa A, Hagberg GE, Peppe A, Bianciardi M, Gioia MC (2006). Functional changes in the activity of cerebellum and frontostriatal regions during externally and internally timed movement in Parkinson's disease.. Brain Res Bull.

[18] Yu H, Sternad D, Corcos DM, Vaillancourt DE (2007). Role of hyperactive cerebellum and motor cortex in Parkinson's disease.. Neuroimage.

[19] Jahanshahi M, Jones CR, Zijlmans J, Katzenschlager R, Lee L (2010). Dopaminergic modulation of striato-frontal connectivity during motor timing in Parkinson's disease.. Brain.

[20] Harrington DL, Boyd LA, Mayer AR, Sheltraw DM, Lee RR (2004). Neural representation of interval encoding and decision making.. Cogn Brain Res.

[21] Rao SM, Mayer AR, Harrington DL (2001). The evolution of brain activation during temporal processing.. Nat Neurosci.

[22] Coull JT, Nazarian B, Vidal F (2008). Timing, storage, and comparison of stimulus duration engage discrete anatomical components of a perceptual timing network.. J Cogn Neurosci.

[23] Owen AM, James M, Leigh PN, Summers BA, Marsden CD (1992). Fronto-striatal cognitive deficits at different stages of Parkinson's disease.. Brain.

[24] Lewis SJ, Dove A, Robbins TW, Barker RA, Owen AM (2003). Cognitive impairments in early Parkinson's disease are accompanied by reductions in activity in frontostriatal neural circuitry.. J Neurosci.

[25] Cools R (2006). Dopaminergic modulation of cognitive function-implications for l-DOPA treatment in Parkinson's disease.. Neurosci Biobehav Rev.

[26] Razmy A, Lang AE, Shapiro CM (2004). Predictors of impaired daytime sleep and wakefulness in patients with Parkinson disease treated with older (ergot) vs newer (nonergot) dopamine agonists.. Arch Neurol.

[27] Worsley KJ, Marrett S, Neelin P, Vandal AC, Friston KJ (1996). A unified statistical approach for determining significant signals in images of cerebral activation.. Hum Brain Mapp.

[28] Velanova K, Lustig C, Jacoby LL, Buckner RL (2007). Evidence for frontally mediated controlled processing differences in older adults.. Cereb Cortex.

[29] Ramsey JD, Hanson SJ, Hanson C, Halchenko YO, Poldrack RA (2010). Six problems for causal inference from fMRI.. Neuroimage.

[30] Friston KJ, Buechel C, Fink GR, Morris J, Rolls E (1997). Psychophysiological and modulatory interactions in neuroimaging.. Neuroimage.

[31] Segonne F, Dale AM, Busa E, Glessner M, Salat D (2004). A hybrid approach to the skull stripping problem in MRI.. Neuroimage.

[32] Fischl B, Salat DH, Busa E, Albert M, Dieterich M (2002). Whole brain segmentation: automated labeling of neuroanatomical structures in the human brain.. Neuron.

[33] Sled JG, Zijdenbos AP, Evans AC (1998). A nonparametric method for automatic correction of intensity nonuniformity in MRI data.. IEEE Trans Med Imaging.

[34] Fischl B, Liu A, Dale AM (2001). Automated manifold surgery: constructing geometrically accurate and topologically correct models of the human cerebral cortex.. IEEE Trans Med Imaging.

[35] Segonne F, Pacheco J, Fischl B (2007). Geometrically accurate topology-correction of cortical surfaces using nonseparating loops.. IEEE Trans Med Imaging.

[36] Ferrandez AM, Hugueville L, Lehericy S, Poline JB, Marsault C (2003). Basal ganglia and supplementary motor area subtend duration perception: an fMRI study.. Neuroimage.

[37] Nenadic I, Gaser C, Volz HP, Rammsayer T, Hager F (2003). Processing of temporal information and the basal ganglia: new evidence from fMRI.. Exp Brain Res.

[38] Pouthas V, George N, Poline JB, Pfeuty M, Vandemoorteele PF (2005). Neural network involved in time perception: An fMRI study comparing long and short interval estimation.. Hum Brain Mapp.

[39] McNab F, Klingberg T (2008). Prefrontal cortex and basal ganglia control access to working memory.. Nat Neurosci.

[40] Alexander GE, DeLong MR, Strick PL, Cowan WM (1986). Parallel organization of functionally segregated circuits linking basal ganglia and cortex.. Annual Review of Neuroscience.

[41] Livesey AC, Wall MB, Smith AT (2007). Time perception: manipulation of task difficulty dissociates clock functions from other cognitive demands.. Neuropsychologia.

[42] Chen SH, Desmond JE (2005). Cerebrocerebellar networks during articulatory rehearsal and verbal working memory tasks.. Neuroimage.

[43] Augustine JR (1996). Circuitry and functional aspects of the insular lobe in primates including humans.. Brain Res Brain Res Rev.

[44] Kosillo P, Smith AT (2010). The role of the human anterior insular cortex in time processing.. Brain Struct Funct.

[45] Wittmann M, Simmons AN, Aron JL, Paulus MP (2010). Accumulation of neural activity in the posterior insula encodes the passage of time.. Neuropsychologia.

[46] Menon V, Uddin LQ (2010). Saliency, switching, attention and control: a network model of insula function.. Brain Struct Funct.

[47] Sterzer P, Kleinschmidt A (2010). Anterior insula activations in perceptual paradigms: often observed but barely understood.. Brain Struct Funct.

[48] Melgire M, Ragot R, Samson S, Penney TB, Meck WH (2005). Auditory/visual duration bisection in patients with left or right medial-temporal lobe resection.. Brain Cogn.

[49] Buckner RL, Andrews-Hanna JR, Schacter DL (2008). The brain's default network: anatomy, function, and relevance to disease.. Ann N Y Acad Sci.

[50] Delaveau P, Salgado-Pineda P, Fossati P, Witjas T, Azulay JP (2010). Dopaminergic modulation of the default mode network in Parkinson's disease.. Eur Neuropsychopharmacol.

[51] Van ET, Monchi O, Ballanger B, Strafella AP (2009). Dysfunction of the default mode network in Parkinson disease: a functional magnetic resonance imaging study.. Arch Neurol.

[52] Argyelan M, Carbon M, Ghilardi MF, Feigin A, Mattis P (2008). Dopaminergic suppression of brain deactivation responses during sequence learning.. J Neurosci.

[53] Harrington DL, Lee RR, Boyd LA, Rapcsak SZ, Knight RT (2004). Does the representation of time depend on the cerebellum?: Effect of cerebellar stroke.. Brain.

[54] Graham S, Phua E, Soon CS, Oh T, Au C (2009). Role of medial cortical, hippocampal and striatal interactions during cognitive set-shifting.. Neuroimage.

[55] Helmich RC, Derikx LC, Bakker M, Scheeringa R, Bloem BR (2009). Spatial Remapping of Cortico-striatal Connectivity in Parkinson's Disease.. Cereb Cortex.

[56] Costa RM, Lin SC, Sotnikova TD, Cyr M, Gainetdinov RR (2006). Rapid alterations in corticostriatal ensemble coordination during acute dopamine-dependent motor dysfunction.. Neuron.

[57] Folstein MF, Folstein SE, McHugh PR (1975). Mini-Mental State.. Journal of Psychiatric Research.

[58] Reitan RM, Wolfson D (1993). The Halstead-Reitan Neuropsychological Test Battery: Theory and clinical interpretation..

[59] Wechsler D (1997). Wechsler Adult Intelligence Scale - III..

[60] Kelland DZ, Lewis RF (1996). The Digit Vigilance Test: reliability, validity, and sensitivity to diazepam.. Arch Clin Neuropsychol.

[61] Schmahmann JD, Doyon J, Toga AW, Petrides M, Evans AC (2000). MRI Atlas of the Human Cerebellum..

