# Supplementary material for: Neurobehavioral Mechanisms of Temporal Processing Deficits in Parkinson's Disease
Source: PLoS One. 2011 Feb 25;6(2):e17461. doi: 10.1371/journal.pone.0017461 (PMC3045463; doi:10.1371/journal.pone.0017461)
Supplement: Figure S1 — Regions (red) showing significant task-related activation during the encoding phase in analyses conducted separately for each of the three groups. Brain activation is projected onto the lateral and medial surfaces of the left (rows 1 and 2) and right hemispheres (rows 3 and 4), the anterior (row 5) and posterior (row 6) surfaces of the cerebellum, and sagittal sections of the left (row 7) and right (row 8) basal ganglia. See Table S1 for details about individual activation foci. (DOC) [file pone.0017461.s001.doc]

**
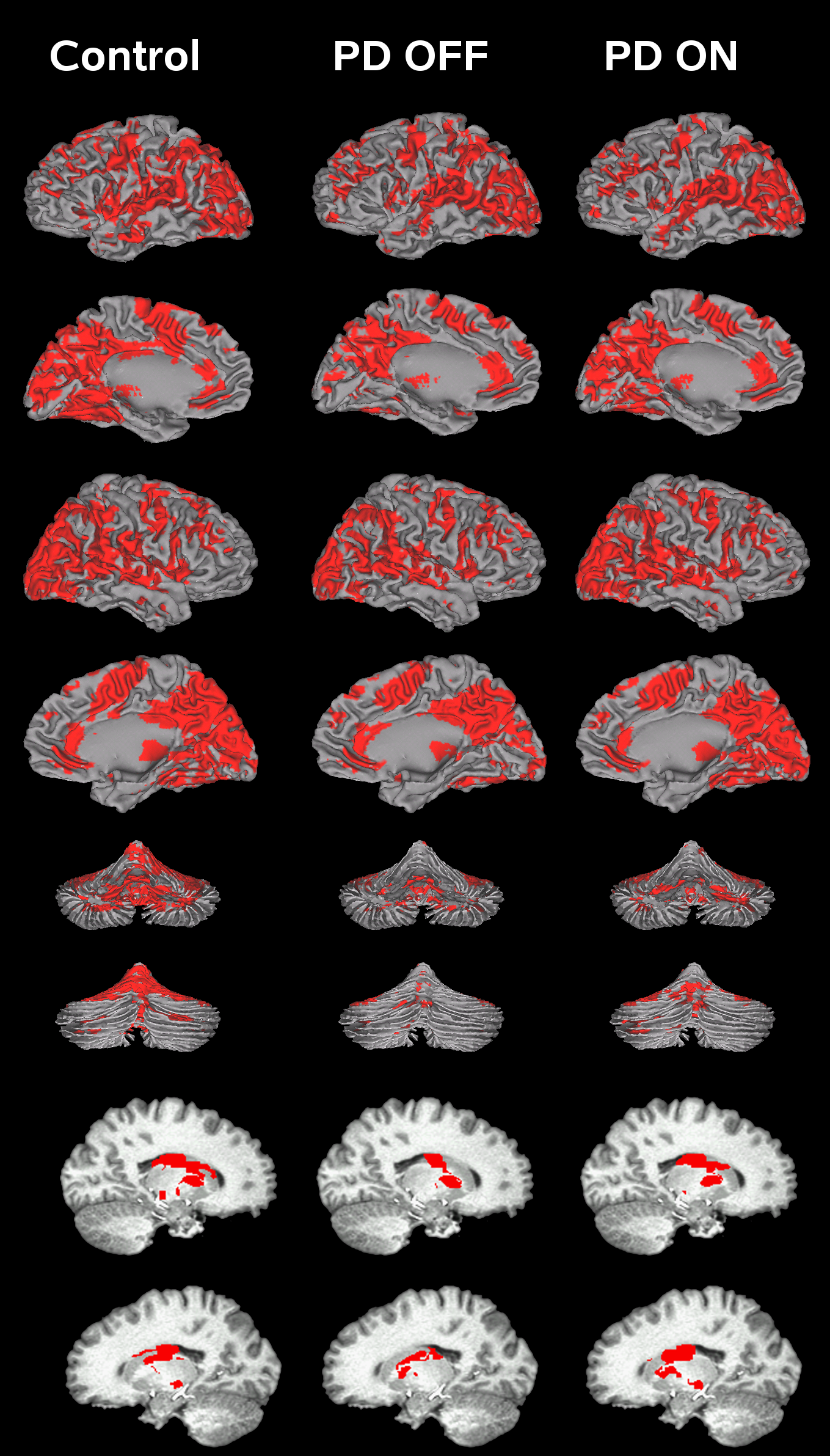
**

**Supplementary Figure 1**. Regions (red) showing significant task-related activation during the encoding phase in analyses conducted separately for each of the three groups. Brain activation is projected onto the lateral and medial surfaces of the left (rows 1 and 2) and right hemispheres (rows 3 and 4), the anterior (row 5) and posterior (row 6) surfaces of the cerebellum, and sagittal sections of the left (row 7) and right (row 8) basal ganglia. See Supplementary Table 1 for details about individual activation foci.
