# Supplementary material for: Neurobehavioral Mechanisms of Temporal Processing Deficits in Parkinson's Disease
Source: PLoS One. 2011 Feb 25;6(2):e17461. doi: 10.1371/journal.pone.0017461 (PMC3045463; doi:10.1371/journal.pone.0017461)
Supplement: Figure S2 — Regions (red) showing significant task-related activation during the decision phase in analyses conducted separately for each of the three groups. Brain activation is projected onto the lateral and medial surfaces of the left (rows 1 and 2) and right hemispheres (rows 3 and 4), the anterior (row 5) and posterior (row 6) surfaces of the cerebellum, and the left (row 7) and right (row 8) basal ganglia. See Table S2 for details about individual activation foci. (DOC) [file pone.0017461.s002.doc]

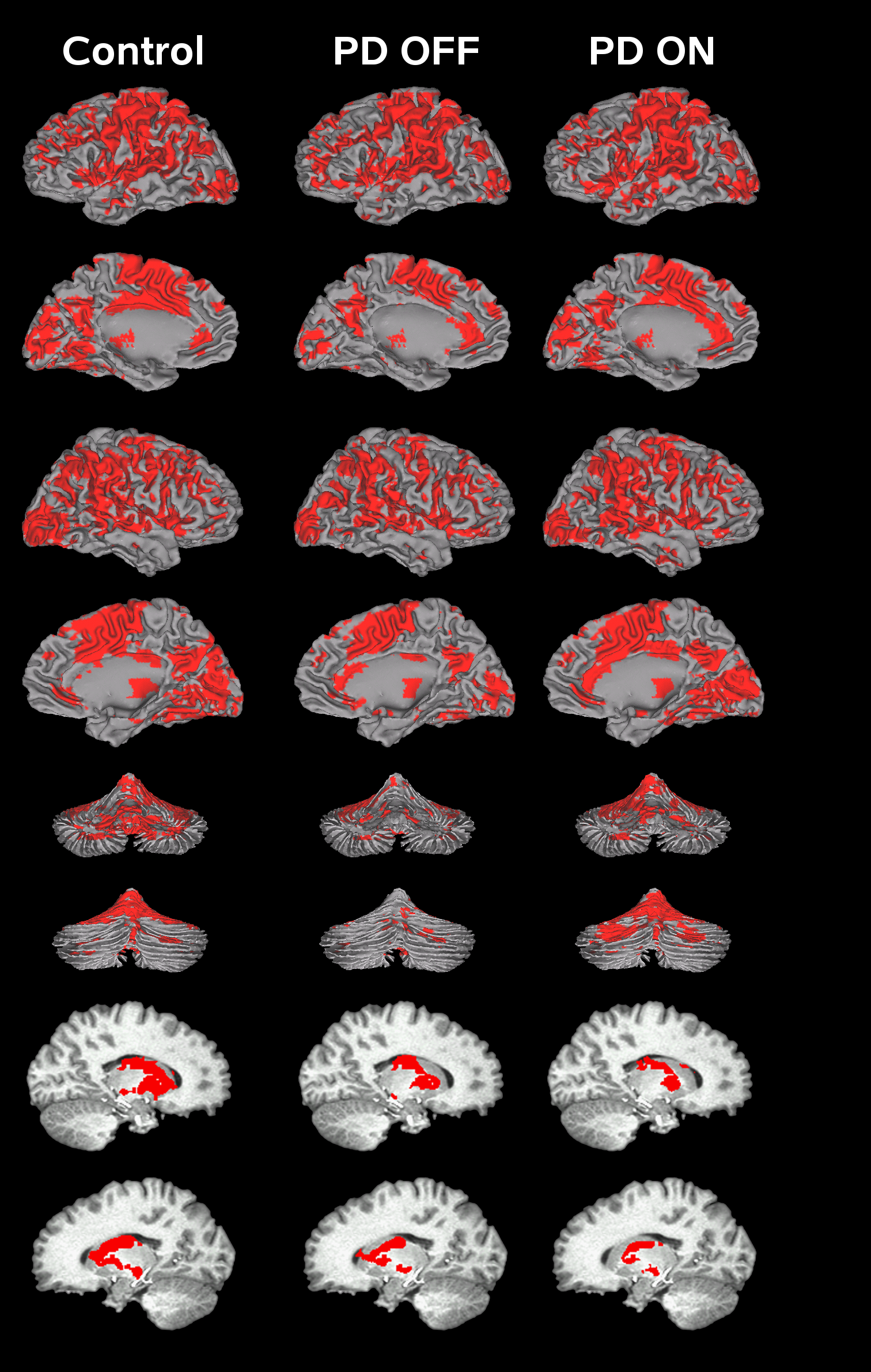


**Supplementary Figure 2**. Regions (red) showing significant task-related activation during the decision phase in analyses conducted separately for each of the three groups. Brain activation is projected onto the lateral and medial surfaces of the left (rows 1 and 2) and right hemispheres (rows 3 and 4), the anterior (row 5) and posterior (row 6) surfaces of the cerebellum, and the left (row 7) and right (row 8) basal ganglia. See Supplementary Table 2 for details about individual activation foci.
