# Supplementary material for: Neurobehavioral Mechanisms of Temporal Processing Deficits in Parkinson's Disease
Source: PLoS One. 2011 Feb 25;6(2):e17461. doi: 10.1371/journal.pone.0017461 (PMC3045463; doi:10.1371/journal.pone.0017461)
Supplement: Table S1 — Activation foci for each group during the encoding phase of a trial. (DOC) [file pone.0017461.s003.doc]

| Table S1. Activation foci for each group during the encoding phase of a trial. | | | | | | | | | | | | | |
| --- | --- | --- | --- | --- | --- | --- | --- | --- | --- | --- | --- | --- | --- |
|  |  | **Control** | | | | **PD OFF** | | | | **PD ON** | | | |
| **Region** | **BA** | **X** | **Y** | **Z** | **μl** | **X** | **Y** | **Z** | **μl** | **X** | **Y** | **Z** | **μl** |
| **Frontal** |  |  |  |  |  |  |  |  |  |  |  |  |  |
| L preSMA/SMA, cingulate | 6,31 | -8 | -6 | 45 | 21277 | -6  -5  -9  -15 | -2  -36  25  -8 | 54  34  52  29 | 6645  4801  957  646 | -6  -6  -15 | -1  -38  -8 | 52  34  29 | 7282  4229  841 |
| R preSMA/SMA, cingulate | 6,31 | 9  7 | 3  -34 | 48  33 | 17313  6553 | 8  7  10 | 4  -35  22 | 52  34  57 | 9633  6836  869 | 8  7  10  18 | 3  -38  25  -6 | 51  34  56  29 | 11672  5332  383  927 |
| B Anterior cingulate | 24 | 3 | 35 | 6 | 10974 | 1 | 35 | 9 | 11466 | 1 | 36 | 7 | 10437 |
| L Precentral | 4,6 | -45  -39 | -6  22 | 35  35 | 12958  382 | -42 | -8 | 37 | 12860 | -39  -50 | -7  0 | 44  10 | 8891  2026 |
| R Precentral | 4,6 | 45 | -3 | 33 | 11554 | 44  28  53 | -4  -25  -7 | 36  60  10 | 8672  1364  863 | 43 | -8 | 38 | 12305 |
| L Superior/middle  (medial, lateral) | 6,8,9,10 | -34  -18  -31  -11  -32 | 35  27  -2  54  40 | 27  48  51  33  -11 | 7492  6801  3362  645  345 | -36  -15  -34 | 24  38  52 | 32  40  -6 | 11832  9864  1372 | -38  -12  -31 | 26  41  54 | 30  40  -1 | 11791  6484  668 |
| R Superior/middle  (medial, lateral) | 6,8,9,10,46 | 35  18 | 18  28 | 38  48 | 16149  4886 | 39  16  33 | 22  40  50 | 33  39  -1 | 11203  9082  697 | 38  15  31 | 20  39  54 | 34  41  0 | 12149  6637  1259 |
| L Inferior | 9,46,47 | -45  -44 | 5  26 | 26  -2 | 2052  1739 | -46 | 6 | 25 | 2504 | -46  -34  -50 | 6  25  27 | 27  -5  11 | 2259  629  587 |
| R Inferior | 9,10,44,45,47 | 48 | 10 | 20 | 4382 | 48  46 | 11  38 | 20  1 | 4718  369 | 47  34 | 12  28 | 19  -7 | 4851  1119 |
| **Parietal** |  |  |  |  |  |  |  |  |  |  |  |  |  |
| L Postcentral | 2,3 | -53 | -18 | 26 | 4935 | -54  -33 | -18  -32 | 20  55 | 2953  3101 | -56 | -19 | 18 | 1973 |
| R Postcentral | 3,5 | 57  23  47 | -18  -39  -17 | 21  64  47 | 3359  944  645 | 57  22  39 | -19  -33  -26 | 18  61  51 | 1769  1503  575 | 57  38 | -20  -26 | 18  52 | 1675  4190 |
| L Superior/precuneus | 7 | -15 | -63 | 40 | 22914 | -15 | -61 | 38 | 16485 | -15 | -63 | 39 | 17577 |
| R Superior/precuneus | 7 | 16 | -63 | 40 | 28034 | 15 | -61 | 39 | 21592 | 15 | -62 | 38 | 19601 |
| L Inferior | 40 | -45  -40 | -42  -68 | 37  32 | 16087  870 | -45  -44 | -41  -68 | 39  32 | 13776  843 | -45  -42 | -43  -70 | 36  32 | 9843  708 |
| R Inferior | 40 | 47 | -45 | 36 | 15245 | 47  48 | -45  -66 | 37  31 | 11709  630 | 47  45 | -44  -69 | 37  31 | 11323  369 |
| B Posterior cingulate |  | 1.4 | -54 | 15 | 12027 | 2 | -53 | 16 | 10157 | 2 | -54 | 15 | 10969 |
| **Temporal** |  |  |  |  |  |  |  |  |  |  |  |  |  |
| L Superior | 22 | -51 | -26 | 8 | 17729 | -51  -38 | -30  8 | 10  -19 | 16142  1168 | -51 | -27 | 8 | 18155 |
| R Superior | 22 | 52 | -28 | 8 | 19601 | 53  41 | -30  5 | 10  -13 | 17310  386 | 53 | -27 | 8 | 18683 |
| L Middle | 21,39 | -45  -55 | -60  -11 | 14  -11 | 8596  3558 | -47  -54 | -60  -11 | 13  -10 | 10151  2313 | -46  -52 | -61  -7 | 14  -13 | 9804  3584 |
| R Middle | 21,39 | 50  50 | -49  2 | 8  -24 | 14537  423 | 51 | -46 | 6 | 12516 | 51 | -44 | 5 | 16025 |
| L Inferior, fusiform gyrus | 20,37 | -34  -57  -47 | -60  -15  -67 | -12  -19  0 | 5521  925  563 | -36  -47 | -60  -68 | -13  0 | 3893  582 | -37  -47 | -53  -67 | -12  0 | 4453  610 |
| R Inferior, fusiform gyrus | 20,37 | 38  60 | -55  -15 | -12  -17 | 5604  352 | 37 | -55 | -13 | 3414 | 37 | -59 | -11 | 5572 |
| L parahippocampus | 36 | -21 | -37 | -6 | 4023 | -26 | -37 | -7 | 795 | -22 | -41 | -5 | 2786 |
| R parahippocampus | 36 | 23 | -36 | -6 | 4848 | 33 | -32 | -17 | 485 | 26 | -37 | -8 | 3646 |
| L Insula | 13 | -39 | -9 | 10 | 12400 | -39 | -12 | 11 | 8551 | -40 | -11 | 10 | 9051 |
| R Insula | 13 | 40 | -8 | 10 | 11274 | 42  38 | -23  13 | 14  6 | 4561  2956 | 41 | -7 | 9 | 7852 |
| **Occipital** |  |  |  |  |  |  |  |  |  |  |  |  |  |
| L Occipital | 17,18,19 | -21 | -80 | 9 | 38487 | -26  -11 | -83  -63 | 10  0 | 23827  807 | -22 | -80 | 10 | 32652 |
| R Occipital | 17,18,19 | 22 | -81 | 10 | 39430 | 26  15 | -82  -64 | 9  1 | 22299  687 | 22 | -80 | 8 | 37142 |
| **Subcortical** |  |  |  |  |  |  |  |  |  |  |  |  |  |
| L Thalamus |  | -13 | -20 | 7 | 5366 | -10 | -17 | 8 | 2930 | -11 | -19 | 8 | 3840 |
| R Thalamus |  | 14 | -21 | 8 | 5849 | 12 | -19 | 8 | 3933 | 13 | -20 | 7 | 4765 |
| L Putamen, globus pallidus |  | -23 | -3 | 6 | 5366 | -23 | -3 | 7 | 4706 | -23 | -5 | 7 | 4051 |
| R Putamen, globus pallidus |  | 25 | -4 | 6 | 4004 | 25 | -4 | 6 | 5266 | 25 | -4 | 7 | 4788 |
| L Caudate (body, tail) |  | -19 | -12 | 15 | 4105 | -15  -27 | -7  -36 | 22  10 | 872  228 | -16  -30 | -10  -37 | 21  6 | 1831  370 |
| R Caudate (head, body, tail) |  | 21  10 | -18  21 | 16  1 | 2723  361 | 17 | -10 | 20 | 1001 | 21 | -14 | 16 | 2661 |
| B Brainstem |  | 0 | -27 | -19 | 6163 | 2  7 | -28  -19 | -5  -18 | 1298  606 | 3 | -25 | -11 | 3820 |
| B Midbrain |  | 1 | -18 | -4 | 3277 | 10  -6 | -18  -20 | -2  -2 | 528  356 | 2 | -19 | -5 | 3251 |
| **Cerebellum** |  |  |  |  |  |  |  |  |  |  |  |  |  |
| B Vermis |  | 0  0  0 | -69  -45  -63 | -24  -13  -4 | 1130  1000  379 | -1 | -72 | -21 | 429 | 0 | -73 | -18 | 762 |
| L Lobule 4-6 |  | -19 | -55 | -16 | 23361 | -28 | -59 | -20 | 7379 | -24 | -59 | -17 | 12397 |
| R Lobule 4-6 |  | 21 | -58 | -16 | 20585 | 34  7  5 | -57  -65  -54 | -20  -8  -19 | 5963  487  338 | 26 | -60 | -18 | 10976 |
| L Lobule 7-10 |  | -19 | -58 | -34 | 15346 | -25  -9 | -63  -47 | -33  -35 | 5831  887 | -26  -7 | -62  -48 | -34  -34 | 11180  632 |
| R Lobule 7-10 |  | 21 | -59 | -34 | 14972 | 18  35  9 | -50  -58  -71 | -37  -28  -25 | 3075  1649  380 | 27  4 | -59  -53 | -34  -32 | 7387  941 |
| **Total Activation Volume** |  |  |  |  |  |  |  |  |  |  |  |  |  |
|  |  |  |  |  | 535782 |  |  |  | 383331 |  |  |  | 434653 |

Brodmann areas (BA) were defined by the Talairach and Tournoux (1988) atlas. Cerebellar lobules were defined by the Schmahmann atlas (Schmahmann et al., 2000). Coordinates represent distance in mm from anterior commissure: x, right(+)/left (-); y, anterior (+)/posterior (-); z, superior (+)/inferior (-). ROIs are displayed in Figure 2. B = bilateral, L= left hemisphere; R = right hemisphere; SMA = supplementary motor area.
