# Supplementary material for: Neurobehavioral Mechanisms of Temporal Processing Deficits in Parkinson's Disease
Source: PLoS One. 2011 Feb 25;6(2):e17461. doi: 10.1371/journal.pone.0017461 (PMC3045463; doi:10.1371/journal.pone.0017461)
Supplement: Table S2 — Activation foci for each group during the decision phase of a trial. (DOC) [file pone.0017461.s004.doc]

| Table S2. Activation foci for each group during the decision phase of a trial. | | | | | | | | | | | | | |
| --- | --- | --- | --- | --- | --- | --- | --- | --- | --- | --- | --- | --- | --- |
|  |  | **Control** | | | | **PD OFF** | | | | **PD ON** | | | |
| **Region** | **BA** | **X** | **Y** | **Z** | **μl** | **X** | **Y** | **Z** | **μl** | **X** | **Y** | **Z** | **μl** |
| **Frontal** |  |  |  |  |  |  |  |  |  |  |  |  |  |
| L preSMA/SMA, cingulate | 6,31 | -7  -5 | -2  -50 | 48  29 | 26540  865 | -7  -6  -12  -16 | -2  -48  23  -11 | 52  31  50  28 | 14081  1191  1139  394 | -7  -13  -17 | -5  25  -23 | 48  48  34 | 20552  628  967 |
| R preSMA/SMA, cingulate | 6,31 | 8  6 | 1  -49 | 47  29 | 27841  1135 | 8  7  13 | 3  -50  24 | 48  29  56 | 19209  994  365 | 8 | -3 | 47 | 27734 |
| B Anterior cingulate | 24,32 | 2  -7 | 38  22 | 3  23 | 4999  596 | 0 | 33 | 9 | 7808 | 0 | 35 | 10 | 10526 |
| L Precentral | 4,6 | -41 | -8 | 40 | 21278 | -40 | -8 | 41 | 17286 | -40 | -9 | 40 | 19498 |
| R Precentral | 4,6 | 42 | -6 | 38 | 15872 | 43 | -4 | 36 | 12436 | 42 | -6 | 38 | 14690 |
| L Superior/middle  (medial, lateral) | 6,8,9,10 | -35  -17 | 25  31 | 33  45 | 18348  9244 | -36  -13 | 25  39 | 29  41 | 18450  8262 | -35  -14 | 21  37 | 34  42 | 14094  7564 |
| R Superior/middle  (medial, lateral) | 6,9,10,11 | 30 | 23 | 37 | 30073 | 31 | 27 | 33 | 24289 | 31  44  4 | 25  40  24 | 36  -7  48 | 25421  576  358 |
| L Inferior | 9,44,47 | -45 | 14 | 13 | 5029 | -45 | 17 | 10 | 7737 | -45 | 17 | 8 | 9327 |
| R Inferior | 9,45,47 | 47 | 18 | 12 | 8865 | 46 | 19 | 10 | 10445 | 45 | 19 | 10 | 10826 |
| **Parietal** |  |  |  |  |  |  |  |  |  |  |  |  |  |
| L Postcentral | 2,3 | -41 | -27 | 45 | 16086 | -42 | -27 | 44 | 14812 | -41 | -27 | 45 | 15379 |
| R Postcentral | 2,3 | 51 | -22 | 35 | 8135 | 52  19 | -24  -48 | 33  65 | 5190  698 | 51  8 | -22  -49 | 35  65 | 7187  346 |
| L Superior/precuneus | 7 | -24  -6  -8  -38 | -58  -57  -71  -72 | 50  30  33  35 | 9981  2004  439  415 | -25  -6 | -58  -56 | 52  33 | 7026  3757 | -24  -7 | -56  -57 | 52  31 | 6958  3265 |
| R Superior/precuneus | 7 | 31  8 | -61  -64 | 46  33 | 6121  5297 | 35  6.4  16 | -58  -56  -54 | 50  31  60 | 1554  2857  1473 | 34  7.4 | -57  -57 | 48  31 | 1482  3785 |
| L Inferior | 40 | -44 | -42 | 38 | 18958 | -47  -43 | -39  -70 | 38  32 | 19875  640 | -46  -42 | -39  -71 | 37  32 | 19374  468 |
| R Inferior | 40 | 47 | -43 | 37 | 20933 | 48  46 | -42  -66 | 38  31 | 16798  347 | 48 | -41 | 38 | 19027 |
| B Posterior cingulate | 23,29,30 | 2  1 | -55  -33 | 15  24 | 6792  480 | 1  19 | -53  -65 | 17  11 | 4000  593 | 5 | -55 | 15 | 5754 |
| **Temporal** |  |  |  |  |  |  |  |  |  |  |  |  |  |
| L Superior | 22 | -51 | -26 | 9 | 18285 | -52  -41 | -26  12 | 8  -28 | 16895  362 | -51  -42 | -26  10 | 8  -23 | 16968  694 |
| R Superior | 22 | 53  52 | -26  -59 | 8  22 | 15962  497 | 53  49 | -24  -57 | 7  21 | 15164  717 | 53 | -24 | 8 | 16006 |
| L Middle | 21,39 | -53  -41 | -29  -67 | -3  22 | 5959  3793 | -56  -39  -51 | -43  -71  -4 | 2  23  -15 | 3468  2332  2502 | -55  -40 | -24  -69 | -6  23 | 8157  2222 |
| R Middle | 21,39 | 54  47  58 | -45  -65  -14 | 2  23  -9 | 5735  2617  1335 | 55  45  55 | -39  -67  -8 | 1  20  -13 | 4194  3393  1742 | 57  46 | -31  -67 | -4  23 | 9243  1743 |
| L Inferior, fusiform gyrus | 20,37 | -33  -57  -46 | -68  -14  -66 | -12  -19  0 | 2648  614  416 |  |  |  |  | -39  -55  -45  -28 | -54  -10  -69  -84 | -12  -20  0  -13 | 1372  989  370  918 |
| R Inferior, fusiform gyrus | 20,37 | 37  48 | -59  -68 | -12  0 | 3703  510 | 35 | -49 | -13 | 726 | 37  46  41  50 | -45  -66  -71  -8 | -13  0  -12  -26 | 1618  390  1677  611 |
| L parahippocampus, amygdala | 36 | -19  -25 | -46  -13 | 3  -12 | 542  512 | -28 | -3 | -14 | 363 | -30  -26 | -35  -7 | -8  -14 | 596  646 |
| R parahippocampus, amygdala | 36 | 23  23 | -47  -23 | -6  -17 | 596  509 |  |  |  |  | 33  28  13 | -32  -5  -45 | -13  -14  3 | 999  460  368 |
| L Insula | 13 | -39 | -7 | 10 | 13520 | -39 | -7 | 9 | 13380 | -39 | -7 | 10 | 13465 |
| R Insula | 13 | 40 | -7 | 10 | 12457 | 40 | -5 | 10 | 9916 | 41 | -5 | 9 | 11150 |
| **Occipital** |  |  |  |  |  |  |  |  |  |  |  |  |  |
| L Occipital | 17,18,19 | -19  -34 | -78  -80 | 2  29 | 21505  977 | -27  -14  -9 | -85  -76  -95 | 4  2  20 | 7929  1673  435 | -31  -10  -2 | -84  -76  -77 | 1  7  31 | 10431  9121  479 |
| R Occipital | 17,18,19 | 21 | -80 | 7 | 32443 | 22 | -83 | 3 | 17883 | 20 | -79 | 6 | 27660 |
| **Subcortical** |  |  |  |  |  |  |  |  |  |  |  |  |  |
| L Thalamus |  | -12 | -18 | 8 | 6794 | -11 | -16 | 9 | 4771 | -11 | -17 | 9 | 5076 |
| R Thalamus |  | 13 | -19 | 8 | 6615 | 11 | -16 | 9 | 3854 | 13 | -18 | 9 | 4843 |
| L Putamen, globus pallidus |  | -22 | -1 | 4 | 8492 | -24 | -3 | 5 | 7812 | -22 | 0 | 5 | 4519 |
| R Putamen, globus pallidus |  | 23 | -1 | 6 | 6111 | 25 | -3 | 5 | 6721 | 24 | -1 | 5 | 4581 |
| L Caudate ((body, tail) |  | -15 | -3 | 13 | 5883 | -14 | -6 | 17 | 2631 | -13 | -9 | 18 | 2191 |
| R Caudate (body, tail, head) |  | 14 | 0 | 16 | 4494 | 14 | 2 | 15 | 2701 | 14 | -5 | 16 | 1443 |
| B Brainstem |  | 0 | -25 | -16 | 5479 | -2  -1 | -22  -27 | -12  -31 | 2364  744 | -1 | -24 | -13 | 3527 |
| B Midbrain |  | 1 | -18 | -4 | 3831 | 1 | -18 | -4 | 2795 | 1 | -17 | -5 | 3492 |
| **Cerebellum** |  |  |  |  |  |  |  |  |  |  |  |  |  |
| B Vermis |  | 1  2  0 | -71  -45  -63 | -24  -13  -3 | 1056  953  571 |  |  |  |  | 0  1  2 | -71  -63  -44 | -22  -4  -11 | 1167  416  397 |
| L Lobule 4-6 |  | -19 | -57 | -17 | 21853 | -24 | -56 | -22 | 5037 | -17 | -59 | -16 | 14383 |
| R Lobule 4-6 |  | 19 | -56 | -16 | 27685 | 23  17 | -52  -30 | -18  -16 | 12827  379 | 18 | -55 | -16 | 24120 |
| L Lobule 7-10 |  | -22 | -59 | -33 | 16605 | -25 | -60 | -33 | 5190 | -24 | -71 | -32 | 9052 |
| R Lobule 7-10 |  | 22 | -59 | -33 | 17904 | 19  28 | -57  -77 | -34  -30 | 4421  1396 | 21 | -65 | -33 | 10484 |
| **Total Activation Volume** |  |  |  |  |  |  |  |  |  |  |  |  |  |
|  |  |  |  |  | 545787 |  |  |  | 390423 |  |  |  | 473830 |

Brodmann areas (BA) were defined by the Talairach and Tournoux (1988) atlas. Cerebellar lobules were defined by the Schmahmann atlas (Schmahmann et al., 2000). Coordinates represent distance in mm from anterior commissure: x, right(+)/left (-); y, anterior (+)/posterior (-); z, superior (+)/inferior (-). ROIs are displayed in Figure ?. B = bilateral, L= left hemisphere; R = right hemisphere; SMA = supplementary motor area.
