# Supplementary material for: Neurobehavioral Mechanisms of Temporal Processing Deficits in Parkinson's Disease
Source: PLoS One. 2011 Feb 25;6(2):e17461. doi: 10.1371/journal.pone.0017461 (PMC3045463; doi:10.1371/journal.pone.0017461)
Supplement: Table S3 — Functional ROI resulting from the conjunction of activations in the Control, PD OFF, and PD On Groups. (DOC) [file pone.0017461.s005.doc]

| **Table 2. Functional ROI resulting from the conjunction of activations in the Control, PD OFF, and PD ON groups ^1^.** | | | | | | | | | | | |
| --- | --- | --- | --- | --- | --- | --- | --- | --- | --- | --- | --- |
| Table S3. Functional ROI resulting from the conjunction of activations in the Control, PD OFF, and PD On  Groups ^1^.  **Encoding Phase Decision Phase** | | | | | | | | | | | |
| **Region** | **BA** | **X** | **Y** | **Z** | **ml** | **Region** | **BA** | **X** | **Y** | **Z** | **ml** |
| **Frontal** |  |  |  |  |  | **Frontal** |  |  |  |  |  |
| L preSMA/SMA, cingulate | 6,31 | -8 | -7 | 45 | 24.1 | L Pre SMA/SMA, cingulate | 6,31 | -8 | -5 | 46 | 30.3 |
| R preSMA/SMA, cingulate | 6,31 | 9 | -7 | 44 | 27.6 | R Pre SMA/SMA, cingulate | 6,31 | 8 | -3 | 46 | 32.6 |
| B Anterior cingulate | 24 | 2 | 35 | 8 | 14.0 | B Anterior cingulate | 24,32 | 1 | 34 | 9 | 12.9 |
| L Precentral | 4,6 | -43 | -7 | 37 | 17.5 | L Precentral | 4,6 | -41 | -8 | 39 | 21.9 |
| R Precentral | 4,6 | 42 | -8 | 38 | 15.9 | R Precentral | 4,6 | 42 | -7 | 39 | 16.6 |
| L Superior/middle  (medial, lateral) | 6,8,9  10 | -28  -33 | 28  51 | 37  -6 | 29.8  2.1 | L Superior/middle  (medial, lateral) | 6,9,10  6,8 | -35  -16 | 27  34 | 29  43 | 23.5  12.0 |
| R Superior/middle  (medial, lateral) | 6,9,10,46  6,9  10 | 36  16  31 | 19  37  52 | 37  41  -1 | 17.7  11.3  1.5 | R Superior/middle  (medial, lateral) | 6,9,10,11 | 30 | 27 | 34 | 37.4 |
| L Inferior | 46,47  9 | -42  -47 | 26  6 | 0  25 | 3.0  2.9 | L Inferior | 9,44,47 | -44 | 19 | 7 | 11.4 |
| R Inferior | 9,44,45,47  10  47 | 48  34  45 | 13  28  40 | 18  -7  1 | 5.8  1.1  .4 | R Inferior | 9,45,47 | 45 | 20 | 8 | 12.6 |
| **Parietal** |  |  |  |  |  | **Parietal** |  |  |  |  |  |
| L Postcentral | 2,3 | -45 | -23 | 37 | 9.0 | L Postcentral | 2,3 | -41 | -26 | 44 | 17.1 |
| R Postcentral | 3,5 | 33  56 | -29  -18 | 55  22 | 6.0  3.5 | R Postcentral | 2,3  5 | 50  16 | -23  -48 | 37  65 | 9.5  .9 |
| L Superior, precuneus | 7 | -15 | -62 | 40 | 24.6 | L Superior, precuneus | 7 | -23  -7  -39 | -58  -58  -72 | 50  33  35 | 10.6  4.6  .5 |
| R Superior, precuneus | 7 | 16 | -62 | 41 | 29.7 | R Superior, precuneus | 7 | 28  8 | -59  -62 | 49  33 | 7.8  6.8 |
| L Inferior | 40 | -46  -43 | -41  -68 | 37  32 | 19.0  1.2 | L Inferior | 40 | -46 | -41 | 37 | 23.5 |
| R Inferior | 40 | 48 | -45 | 36 | 17.3 | R Inferior | 40 | 47 | -43 | 37 | 22.3 |
| B Posterior cingulate | 23 | 1 | -54 | 15 | 13.4 | B Posterior cingulate | 29,30  23 | 3  1 | -55  -33 | 14  24 | 8.8  .5 |
| **Temporal** |  |  |  |  |  | **Temporal** |  |  |  |  |  |
| L Superior | 22 | -50 | -24 | 6 | 21.7 | L Superior | 22 | -51 | -24 | 7 | 21.2 |
| R Superior | 22 | 52 | -27 | 8 | 20.8 | R Superior | 22 | 52  50 | -24  -58 | 7  22 | 17.6  .9 |
| L Middle | 39  21 | -47  -54 | -59  -9 | 13  -13 | 12.3  4.7 | L Middle | 21  39 | -54  -41 | -28  -67 | -5  22 | 11.0  4.3 |

| R Middle | 21,39 | 51 | -45 | 5 | 18.4 | R Middle | 21,39 | 53 | -42 | 3 | 16.8 |
| --- | --- | --- | --- | --- | --- | --- | --- | --- | --- | --- | --- |
| L Inferior, fusiform gyrus | 37  20  37 | -35  -57  -47 | -59  -15  -67 | -12  -19  0 | 6.3  1.0  .6 | L Inferior, fusiform gyrus | 37  20  37 | -35  -54  -46 | -66  -11  -67 | -13  -21  0 | 3.5  1.3  .5 |
| R Inferior, fusiform gyrus | 37  20 | 38  60 | -59  -14 | -12  -17 | 7.2  .4 | R Inferior, fusiform gyrus | 37  20  37 | 39  50  48 | -59  -8  -67 | -12  -26  0 | 5.5  .6  .6 |
| L parahippocampus. | 36 | -22 | -39 | -6 | 5.0 | L parahippocampus, amygdala | 36 | -28  -19 | -19  -46 | -12  3 | 1.8  .5 |
| R parahippocampus | 36 | 25 | -36 | -7 | 6.0 | R parahippocampus, amygdala | 36 | 27  14  28 | -33  -45  -5 | -12  3  -14 | 2.3  .5  .5 |
| L Insula | 13 | -39 | -9 | 10 | 12.6 | L Insula | 13 | -39 | -7 | 9 | 14.4 |
| R Insula | 13 | 40 | -8 | 9 | 12.0 | R Insula | 13 | 40 | -7 | 9 | 13.6 |
| **Occipital** |  |  |  |  |  | **Occipital** |  |  |  |  |  |
| L Occipital | 17,18,19 | -20 | -80 | 9 | 43.4 | L Occipital | 17,18,19 | -20 | -80 | 5 | 28.9 |
| R Occipital | 17,18,19 | 21 | -81 | 9 | 45.2 | R Occipital | 17,18,19 | 21 | -80 | 7 | 38.4 |
| **Subcortical** |  |  |  |  |  | **Subcortical** |  |  |  |  |  |
| L Thalamus |  | -13 | -20 | 8 | 5.9 | L Thalamus |  | -12 | -18 | 8 | 6.3 |
| R Thalamus |  | 14 | -20 | 8 | 5.9 | R Thalamus |  | 13 | -19 | 8 | 6.2 |
| L Putamen, GP |  | -24 | -3 | 5 | 7.1 | L Putamen, GP |  | -23 | -2 | 3 | 9.8 |
| R Putamen, GP |  | 25 | -4 | 5 | 6.7 | R Putamen, GP |  | 24 | -2 | 5 | 7.8 |
| L Caudate (body, tail) |  | -20 | -13 | 15 | 4.5 | L Caudate (head, body, tail) |  | -15 | -4 | 13 | 6.4 |
| R Caudate (body, tail)  (head) |  | 21  9 | -16  20 | 16  0 | 3.5  .4 | R Caudate (head, body, tail) |  | 13 | 2 | 15 | 5.1 |
| B Brainstem |  | 1 | -27 | -18 | 7.8 | B Brainstem |  | 0 | -24 | -17 | 7.4 |
| B Midbrain |  | 1 | -18 | -5 | 3.8 | B Midbrain |  | 1 | -17 | -5 | 4.3 |
| **Cerebellum** |  |  |  |  |  | **Cerebellum** |  |  |  |  |  |
| B Vermis (a, p)  (a)  (a) |  | 0  0  0 | -70  -45  -63 | -23  -13  -3 | 1.5  1.0  .5 | B Vermis (a, p)  (a)  (a) |  | 0  1  0 | -71  -45  -63 | -23  -13  -3 | 1.5  1.0  .6 |
| L Lobule 4-6 |  | -19 | -56 | -16 | 24.9 | L Lobule 4-6 |  | -19 | -57 | -17 | 24.1 |
| R Lobule 4-6 |  | 21 | -58 | -17 | 23.0 | R Lobule 4-6 |  | 20 | -56 | -16 | 29.8 |
| L Lobule 7-10 |  | -21 | -60 | -34 | 20.8 | L Lobule 7-10 |  | -22 | -63 | -33 | 21.5 |
| R Lobule 7-10 |  | 23 | -59 | -34 | 19.3 | R Lobule 7-10 |  | 22 | -62 | -33 | 22.3 |
| Total Volume |  |  |  |  | 652.9 | Total Volume |  |  |  |  | 662.7 |
| fROIs are displayed in Figure 3.  ^1^ Conjunction ROIs were constructed separately for activations during the encoding (left columns) and decision (right columns) phases. Brodmann areas (BA) were defined by the Talairach and Tournoux (1988) atlas. Cerebellar lobules were defined by the Schmahmann atlas (Schmahmann et al., 2000). Coordinates represent distance in mm from anterior commissure: x, right(+)/left (-); y, anterior (+)/posterior (-); z, superior (+)/inferior (-). B = bilateral, L= left hemisphere; R = right hemisphere; GP = globus pallidus; SMA = supplementary motor area; a = anterior cerebellar lobule; b = posterior cerebellar lobule. | | | | | | | | | | | |
